# Enhanced nuclear localization of small heterodimer partner in metabolic dysfunction-associated steatohepatitis

**DOI:** 10.1016/j.jhepr.2025.101616

**Published:** 2025-10-04

**Authors:** Shih-Chieh Chien, Chiung-Yu Chen, Hung-Wen Tsai, Yih-Jyh Lin, Shu-Chu Shiesh, Pin-Nan Cheng, Hung-Chih Chiu, Yen-Cheng Chiu, Ya-Han Lin, Min-Shan Wu, Mei-Juan Zheng, Kung-Chia Young, Yau-Sheng Tsai

**Affiliations:** 1 Institute of Clinical Medicine, College of Medicine, National Cheng Kung University, Tainan, Taiwan; 2 Department of Internal Medicine, National Cheng Kung University Hospital, College of Medicine, National Cheng Kung University, Tainan, Taiwan; 3 Department of Pathology, National Cheng Kung University Hospital, College of Medicine, National Cheng Kung University, Tainan, Taiwan; 4 Division of General and Transplant Surgery, Department of Surgery, National Cheng Kung University Hospital, College of Medicine, National Cheng Kung University, Tainan, Taiwan; 5 Department of Medical Laboratory Science and Biotechnology, College of Medicine, National Cheng Kung University, Tainan, Taiwan; 6 Clinical Medicine Research Center, National Cheng Kung University Hospital, College of Medicine, National Cheng Kung University, Tainan, Taiwan

**Keywords:** Metabolic dysfunction-associated steatohepatitis, Bile acids, Small heterodimer partner, Nuclear factor kappa B, Protein kinase C zeta

## Abstract

**Background & Aims:**

The nuclear factor small heterodimer partner (SHP) plays a dual function in maintaining bile acid (BA) homeostasis and exerting anti-inflammatory effects. While SHP might be associated with metabolic dysfunction-associated steatohepatitis (MASH), its role in patients remains unclear.

**Methods:**

Liver tissue and serum samples were collected from 69 patients with MASH and 10 healthy controls. The subcellular distribution of SHP and related proteins in liver tissue were analyzed to correlate with MASH-associated pathological characteristics. *In vitro* studies were conducted to elucidate the underlying mechanisms and clinical relevance of SHP nuclear translocation in MASH.

**Results:**

Compared with controls, patients with MASH demonstrated a higher nuclear SHP ratio (51.7% *vs.* 1.55%, *p* <0.001), which correlated with the severity of hepatitis and steatosis, but not with serum BA levels. The nuclear SHP ratio increased in parallel with atypical protein kinase C zeta (PKCζ) signal intensity and showed high co-localization with nucleoporin RanBP2. *In vitro* experiments demonstrated that both toxic fatty acid and inflammatory cytokine could induce SHP nuclear translocation, which was blocked by PKCζ inhibition. *SHP* knockdown increased basal innate immune activity, accelerated intracellular lipid accumulation, and recapitulated a MASH-like gene profile that facilitates BA accumulation.

**Conclusions:**

Nuclear SHP accumulation is a distinctive feature of MASH pathology. This PKCζ-dependent process may exert anti-inflammation and anti-cholestasis function in patients with MASH.

**Impact and implications:**

The nuclear factor small heterodimer partner (SHP) plays a dual role in maintaining bile acid homeostasis and suppressing inflammation. In patients with metabolic dysfunction-associated steatohepatitis (MASH), we identified a pathological increase in the hepatocellular nuclear SHP ratio, likely triggered by lipid overload and inflammatory stimuli, and dependent on PKCζ activation. *SHP* knockdown *in vitro* induced cellular steatosis, innate immune responses, and leading to a cholestatic gene expression profile. These findings highlight SHP as a potential therapeutic target in MASH.

## Highlights

• Nuclear SHP accumulation is a hallmark of MASH and correlates with steatosis and hepatitis severity.
• PKCζ activation drives SHP nuclear translocation in response to lipid overload and inflammation.
• SHP knockdown increases steatosis, innate immune activation, and cholestatic gene expression.
• Findings identify SHP as a potential therapeutic target for metabolic dysfunction-associated steatohepatitis.

## Graphical abstract

**Figure:**
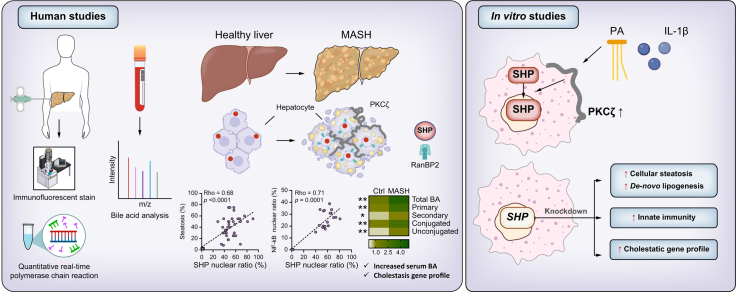

## Introduction

Metabolic dysfunction-associated steatotic liver disease (MASLD) poses a global healthcare burden.^1^ About 16–20% of patients with MASLD progress to metabolic dysfunction-associated steatohepatitis (MASH),^2^ characterized by steatosis and hepatocellular injury.^3^ Patients with MASH exhibit increased risks of cirrhosis and associated comorbidities.^4^^,^^5^

The small heterodimer partner (SHP), an orphan nuclear receptor, acts as a transcriptional repressor for maintaining bile acid (BA) homeostasis.^6^^,^^7^ SHP interacts with downstream targets and represses their transcriptional activities by recruiting co-repressors or interfering with enhancers, leading to the downregulation of target genes, including cytochrome P450 family 7 subfamily A member 1 (*CYP7A1*).[8], [9], [10] Patients with MASH exhibit elevated serum BA levels[11], [12], [13] and increased *CYP7A1* expression in liver tissues,^11^^,^^13^ suggesting a dysregulated SHP-CYP7A1 axis for BA synthesis.

Beyond BA homeostasis, SHP also mitigates liver inflammation.[14], [15], [16], [17], [18] Studies using hepatic-specific SHP-knockout mice show that diet-induced hepatic inflammation is exacerbated without SHP, whereas SHP overexpression reduces this effect.^15^^,^^18^ SHP interacts with nuclear factor kappa B (NF-κB) in inflammatory cells by reducing the phosphorylation of the IκB kinase (IKK) complex, thereby inhibiting NF-κB nuclear translocation and activation.^15^^,^^17^ The repressive function of SHP requires nuclear translocation, which is regulated through phosphorylation by atypical protein kinase C zeta (PKCζ) and SUMOylation by the nucleoporin RAN binding protein 2 (RanBP2).^19^^,^^20^ Disrupted PKCζ-SHP or RanBP2-SHP interactions hinder SHP nuclear translocation in hepatocytes.^19^^,^^20^ PKCζ activation also regulates hepatic steatosis, insulin resistance, and intrahepatic cholestasis.[21], [22], [23], [24], [25] However, the role of SHP in steatosis, inflammation, and BA homeostasis in patients with MASH remains unclear.

In this study, we investigated whether MASH-associated lipotoxicity and inflammation affect SHP protein distribution in liver tissues and explored the correlations between SHP subcellular localizations and MASH pathological parameters, BA composition and synthesis, and PKCζ/RanBP2 distribution. Additionally, using *in vitro* experiments, we examined the role of PKCζ in SHP nuclear translocation and the effect of SHP on hepatic inflammation and BA accumulation.

## Materials and methods

### Inclusion and exclusion criteria for patients with MASH and control participants

Patients were enrolled from the National Cheng Kung University Hospital (NCKUH), Taiwan. Baseline data included abdominal sonography, liver stiffness measurement using vibration-controlled transient elastography (FibroScan, Echosens, Paris, France), laboratory tests, liver tissue analysis, and fasting serum samples. The diagnosis of MASH followed the Delphi consensus^26^ and NASH-Clinical Research Network (NASH-CRN) criteria, requiring a Non-alcoholic fatty liver disease (NAFLD) Activity Score (NAS) ≥3, with at least a score of 1 for steatosis, ballooning, and lobular inflammation, per the fatty liver inhibition of progression algorithm.^3^^,^^27^^,^^28^ Exclusion criteria included individuals with a recent history (within 5 years) of active alcohol consumption or chronic liver diseases other than MASH, including chronic hepatitis B and C, autoimmune hepatitis, primary biliary cholangitis, and Wilson disease. Additionally, patients with a history of primary liver cancer, including hepatocellular carcinoma or intrahepatic cholangiocarcinoma, were excluded. Patients taking medications affecting BA metabolism, inducing hepatic steatosis, or altering MASH course, including fibrate, cholestyramine, pioglitazone, high-dose vitamin E, and glucagon-like peptide-1 agonists, and those in the LiverTox database^29^ were excluded. To acquire healthy liver tissues for comparison, we prospectively collected samples from living liver donors in collaboration with the liver transplantation team at NCKUH and excluded donors with hepatic steatosis or any other liver pathology (Fig. S1). To minimize the influence of circadian rhythm^30^^,^^31^ and feeding effects on SHP activity,^32^^,^^33^ all samples of the participants, including liver tissue and serum, were collected in the morning between 08:30 and 10:30 after overnight fasting. A detailed algorithm for patient selection is presented in Fig. S1. This study was approved by the Institutional Review Board of the NCKUH (IRB number: A-ER-110-503).

### Examination of pathological characteristics

MASH severity was determined using the NAS according to the NASH-CRN criteria,^3^ encompassing steatosis, lobular inflammation, and hepatocellular ballooning, and was reviewed by a specialized pathologist.

### Hepatic lipid quantification

Hepatic lipid droplets were quantified using a semiquantitative method based on H&E staining, a conventional approach used for grading steatosis within the NAS scoring system.^3^ The pathologist estimated the lipid droplet proportion by assessing representative areas under low-power magnification, followed by confirmation with high-power magnification. To improve lipid droplet quantification accuracy, we applied artificial intelligence (AI)-assisted image analysis to liver biopsy specimens.^34^ Results were expressed as the percentage of lipid area relative to the total tissue area for both methods.

### Analysis of serum BA profile

Liquid chromatography–mass spectrometry (API 5000 tandem mass spectrometer, SCIEX, Framingham, MA) was used for BA quantification and analysis. Data were categorized based on the presence of MASH, and MASH severity was further classified according to the NAS, with living donors serving as the control group.

### Analysis of mRNA

Total RNA was extracted from the liver tissue, which was obtained from biopsy, surgery, or cultured cells. mRNA levels were analyzed using quantitative reverse transcriptase-PCR (qRT-PCR; StepOne; Applied Biosystems, Waltham, MA). *GAPDH* or *ACTB* was used as a reference gene for each qRT-PCR reaction. The primer sequences used for qPCR are listed in Table S1.

## Immunofluorescent staining, quantification of human tissue staining, and *in vitro* experiments

The materials and methodological details regarding human tissue immunofluorescent staining, quantification, and *in vitro* experiments used in this study are provided in the Supplementary Materials and methods.

### Statistical analysis

Results were stratified according to the NAS, fibrotic stage, or MASH pathological characteristics. Individuals with relatively healthy livers were designated as the control group. Differences between groups were tested using non-parametric or parametric methods, such as the Kruskal–Wallis test or analysis of variance, with corresponding *post-hoc* analysis. Categorical data were compared using the corresponding Χ^2^ or Fisher’s exact tests. Correlations between variables were evaluated using Spearman’s test. For factors significantly associated with the nuclear SHP ratio, we evaluated their β coefficient using multivariable linear regression. If the factors exhibited multicollinearity, only one was selected for analysis. A *p* value <0.05 was considered statistically significant.

## Results

### Patients with MASH exhibit increased body weight, metabolic dysregulation, and hepatic injury

We prospectively collected samples from 68 patients with biopsy-confirmed MASH and 10 healthy control participants between 2015 and 2020. The median NAS of patients with MASH was 4 (range: 3–7), with 47% exhibiting significant fibrosis (stage: 3–4). Compared with control individuals, patients with MASH were older, with a higher BMI and an increased likelihood of exhibiting features of metabolic syndrome, including a higher prevalence of type II diabetes mellitus, hypertension, and hyperlipidemia. Additionally, they showed elevated levels of serum liver injury markers, including alanine transaminase (ALT), aspartate aminotransferase (AST), alkaline phosphatase (ALP), and gamma-glutamyl transferase (γ-GT). Moreover, these patients exhibited higher serum triglyceride (TG) levels, fasting glucose levels, and insulin resistance, as measured using the Homeostatic Model of Assessment Insulin Resistance (HOMA-IR) (Table S2).

### Patients with MASH exhibit elevated nuclear localization of SHP, which is associated with hepatic steatosis and inflammation

**Fig. 1:**
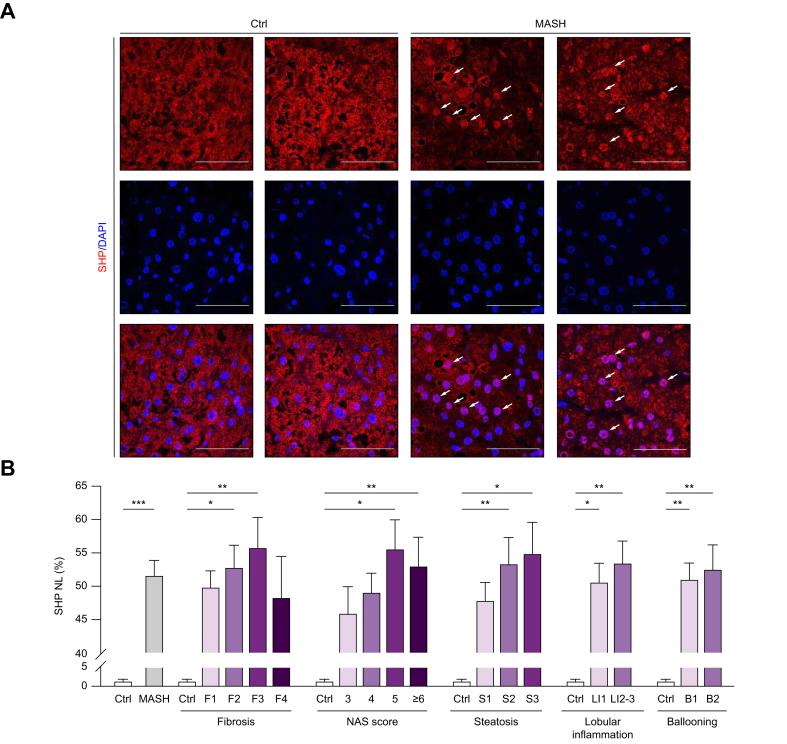
Increased nuclear SHP ratio in patients with MASH is associated with disease severity. (A) Liver samples from patients with MASH showed elevated nuclear SHP signal intensity (white arrows). Scale bar = 50 μm. (B) The SHP NL ratio was significantly higher in patients with MASH, as it increased progressively with disease severity (*p* for trends <0.001, one-way ANOVA). MASH, metabolic dysfunction-associated steatohepatitis; NAS, NAFLD Activity Score; SHP, small heterodimer partner; NL, nuclear localization; ANOVA, Analysis of Variance.

Immunofluorescent staining for SHP was performed on liver biopsy samples. The overall SHP signal intensity did not differ significantly between the specimens of patients with MASH and those of the control participants (Fig. 1A). However, the nuclear SHP ratio was significantly higher in the MASH group compared with the control group (nuclear ratio of MASH *vs.* control group: 51.7% *vs.* 1.55%, *p* = 0.01). Moreover, a stepwise increase in the SHP nuclear ratio was observed in relation to the fibrosis stage, NAS, degree of lobular inflammation, and hepatocyte ballooning (Fig. 1B). We compared the nuclear SHP ratio in patients with low-risk MASH (NAS ≤3 and fibrosis stage ≤2) and those at-risk MASH (NAS ≥4 and fibrosis stage ≥2).^35^^,^^36^ Both groups exhibited similarly elevated nuclear SHP ratios compared with the controls. Additionally, no significant difference in the nuclear SHP ratio was observed between patients with a NAS of 3–4 and those with NAS ≥5 (Fig. S2).

**Fig. 2:**
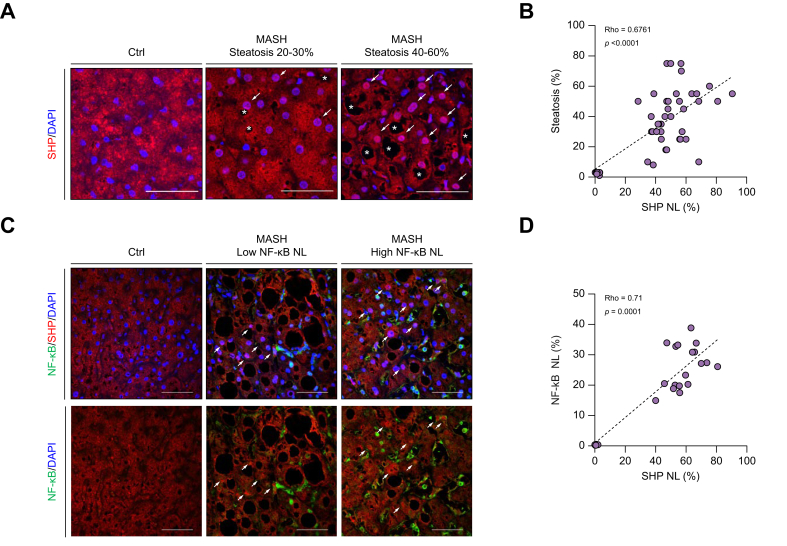
Nuclear SHP signal intensity increases with the extent of tissue steatosis (A and B) and inflammation (C and D, as shown by co-staining of NF-κB and SHP). (Nuclear SHP: arrows, lipid droplet: asterisks; scale bar = 50 μm, Rho: Spearman´s correlation coefficient). MASH, metabolic dysfunction-associated steatohepatitis; NF-κB, nuclear factor kappa B; SHP, small heterodimer partner; SHP NL, nuclear SHP.

SHP nuclear localization was more pronounced in areas with higher lipid accumulation (Fig. 2A) and positively correlated with hepatic steatosis, as assessed using semiquantitative and AI-assisted quantitative methods (Fig. 2B and Fig. S3). Furthermore, NF-κB p65 co-staining revealed an elevated nuclear NF-κB ratio in patients with MASH compared with controls, with predominant nuclear staining observed in the nuclei of inflammatory cells rather than hepatocytes (Fig. 2C). The nuclear ratios of NF-κB and SHP were significantly and positively correlated (Fig. 2D). Taken together, these findings suggest that increased SHP nuclear localization is a key histological feature of MASH and is strongly associated with hepatic steatosis and hepatitis.

IL-1 receptor in hepatocytes acts as a co-factor in metabolic inflammation, liver injury, and hepatocarcinogenesis.^37^ Although *IL1B* mRNA levels were not significantly correlated with the nuclear SHP ratio (Rho = -0.001, *p* = 0.75), they were positively associated with serum ALT and AST levels and were significantly reduced in patients receiving statin therapy (Table S3). These findings suggest that hepatic IL-1β in patients with MASH may be linked to liver injury and influenced by statin use, but does not appear to affect SHP nuclear localization significantly.

### Elevated nuclear SHP localization correlates with key clinical MASH characteristics

To elucidate the clinical significance of elevated hepatic SHP nuclear localization, we assessed its relationship with the clinical parameters in 38 patients with MASH and 10 control individuals with available SHP nuclear ratio data. A significant positive correlation was observed between the nuclear SHP ratio and serum markers of hepatic inflammatory injury, specifically ALT, AST, and γ-GT (Table 1), whereas no correlation was found with ALP and ferritin. Furthermore, the nuclear SHP ratio was significantly positively correlated with lipid metabolic dysregulation, including the degree of hepatic steatosis and fasting serum TG levels. Multivariable linear regression analysis identified serum ALT (β coefficient: 0.12, 95% CI: 0.04–0.20, *p* = 0.006) and the histological degree of hepatic steatosis (β coefficient: 0.35, 95% CI: 0.12–0.59, *p* = 0.004) as the primary independent factors associated with elevated nuclear SHP localization.

**Table 1:** The correlations of nuclear SHP ratio with clinical markers of hepatitis, parameters associated with metabolic dysfunction, and serum BAs.

	Rho	*p* value	β (95% CI)	*p* value
Age	0.28	0.053	0.19 (-0.17, 0.54)	0.29
Sex	0.03	0.82	-2.56 (-12.45, 7.32)	0.60
ALT	0.56	<0.001∗∗∗	0.12 (0.04, 0.20)	0.006∗∗
AST	0.48	0.001∗∗		
γ-GT	0.52	<0.001∗∗∗	0.0004 (-0.05, 0.05)	0.99
ALP	0.25	0.10		
Ferritin	-0.07	0.68		
Steatosis (%)†	0.68	<0.001∗∗∗	0.35 (0.12, 0.59)	0.004∗∗
BMI	0.47	<0.001∗∗∗	0.77 (-0.20, 1.73)	0.12
TG‡	0.49	0.001∗∗	0.05 (-0.0002, 0.09)	0.051
Cholesterol‡	0.24	0.14		
LDL‡	0.10	0.6		
HDL‡	0.07	0.7		
HOMA-IR‡	0.21	0.32		
Insulin‡	0.19	0.37		
HbA1C‡	0.05	0.83		
Fasting glucose‡	0.34	0.049∗	0.17 (-0.05, 0.39)	0.12
Total BA	0.22	0.14		
Conjugated BA	0.19	0.19		
Unconjugated BA	0.24	0.1		
Primary BA	0.22	0.13		
Secondary BA	0.14	0.36		

Rho: Spearman’s correlation coefficient. (∗*p* <0.05, ∗∗*p* <0.01, ∗∗∗*p* <0.001).

γ-GT, gamma-glutamyl transferase; ALP, alkaline phosphatase; ALT, alanine transaminase; AST, aspartate aminotransferase; BMI, body mass index; TG, fasting triglyceride; LDL: low-density lipoprotein; HDL: high density lipoprotein; BA, bile acid; HbA1C, glycosylated hemoglobin A1C; HOMA-IR, Homeostatic Model of Assessment Insulin Resistance; SHP, small heterodimer partner.

† Semiquantitative degree by pathologist.

‡ Only enrolled those not taking medications for type II diabetes mellitus or dyslipidemia.

Although serum glucose level showed a marginal correlation with SHP nuclear localization, glycemic parameters, including serum insulin, HOMA-IR, and glycosylated hemoglobin A1C (HbA1C), were not significantly associated with SHP nuclear localization. Additionally, atherogenic lipid profiles, including total cholesterol, low-density lipoprotein (LDL), and high-density lipoprotein (HDL) levels, were not correlated with the nuclear SHP ratio. SHP nuclear localization did not differ between statin and non-statin users (57.5% *vs.* 49.9%, *p* = 0.27) or between patients with and without type II diabetes mellitus medications (52.3% *vs.* 51.1%, *p* = 0.95) (Table S4). Although control participants were significantly younger, no significant correlation existed between age and nuclear SHP ratio in all participants (Rho = 0.28, *p* = 0.053; Table 1, first row) or in patients with MASH alone (Rho = −0.24, *p* = 0.15; data not shown). These findings suggest that increased SHP nuclear localization is closely associated with serum parameters related to hepatic inflammation and hepatic lipid metabolic dysregulation in patients with MASH.

### BA accumulation is not associated with hepatic SHP nuclear localization

**Fig. 3:**
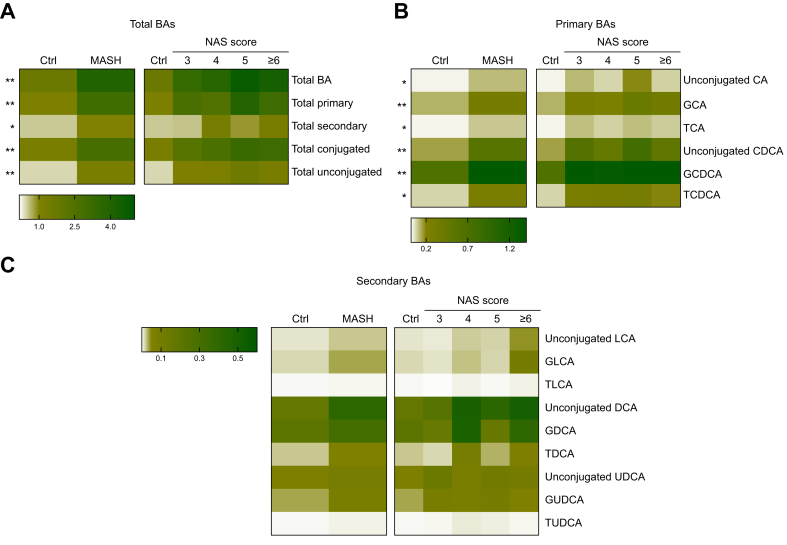
Serum BA profile in the study participants. (A) Serum BA levels were significantly elevated in patients with MASH. (B) Significant increases were observed in the primary and conjugated BA levels. (C) In contrast, an increase in secondary BA levels was relatively insignificant. ∗*p* <0.05, ∗∗*p* <0.01, ∗∗∗*p* <0.001, Mann–Whitney *U* test (for two groups) and Kruskal–Wallis test (for ≥3 groups). BA, bile acid; MASH, metabolic dysfunction-associated steatohepatitis; NAS, NAFLD Activity Score; BA, bile acids; CA, cholic acid; CDCA, chenodeoxycholic acid; LCA, lithocholic acid; DCA, deoxycholic acid; UDCA, ursodeoxycholic acid; G, glyco-; T, tauro-.

To investigate BA homeostasis, serum BA levels were analyzed and visually represented using a heatmap, illustrating the mean values for each group (Fig. 3). Patients with MASH exhibited elevated total serum BA levels, with the most pronounced increase observed in the primary and conjugated BA levels (Fig. 3A and B). Among the primary BAs, the levels of cholic acid (CA), glyco-conjugated CA, chenodeoxycholic acid (CDCA), and tauro-conjugated forms (GCDCA and TCDCA) were significantly increased in the MASH group (Fig. 3B). In contrast, although total secondary BA levels were elevated in patients with MASH, no individual secondary BA showed a significant difference between patients with MASH and control individuals (Fig. 3C). Although DCA is known to drive obesity-induced liver cancer,^38^ no significant differences in DCA levels were observed between patients with MASH and control individuals. Moreover, serum DCA levels did not correlate with hepatitis biomarkers or disease severity (Fig. S4). These findings suggest that patients with MASH exhibit dysregulated primary BA metabolism.

**Fig. 4:**
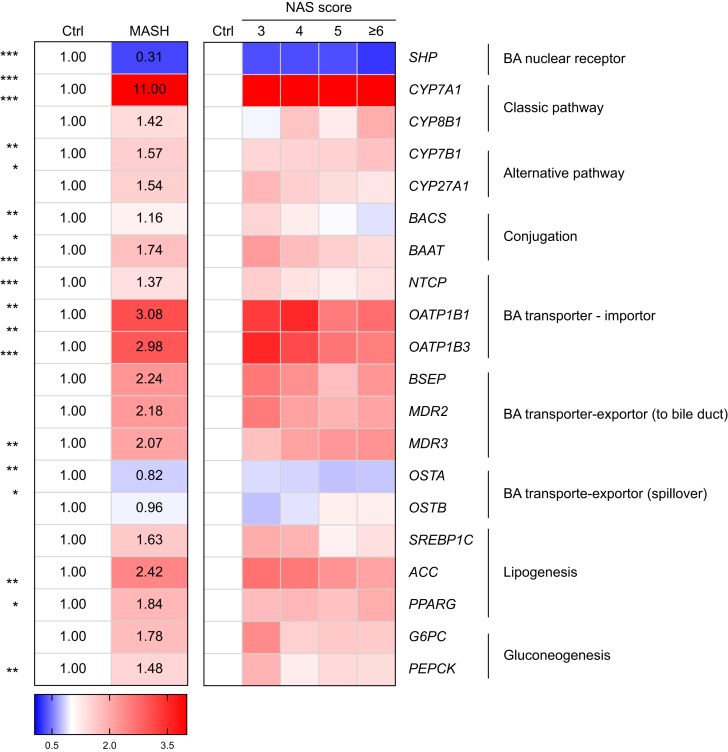
Cholestatic gene profile in patients with MASH. The mRNA profile of BA-related genes in hepatic tissues from patients with MASH favors cholestasis, including a significant increase in genes related to BA synthesis (*CYP7A1*, *CYP7B1*, and *CYP27A1*), conjugation (*BAAT*), and importers (*NTCP*, *OATP1B1*, and *OATP1B3*). The exporter genes (*BSEP*, *MDR2*, and *MDR3*), genes related to lipogenesis (*ACC* and *PPARG*) and gluconeogenesis (*G6PC*) were also increased. ∗*p* <0.05, ∗∗*p* <0.01, ∗∗∗*p* <0.001, Mann–Whitney *U* test (for two groups) and Kruskal–Wallis test (for three or more groups). BA, bile acid; MASH, metabolic dysfunction-associated steatohepatitis; NAS, NAFLD Activity Score; *SHP,* small heterodimer partner; *CYP7A1*, cytochrome P450 family 7 subfamily A member 1; *CYP8B1*, cytochrome P450 family 8 subfamily B member 1; *CYP7B1*, cytochrome P450 family 7 subfamily B member 1, *CYP27A1*, cytochrome P450 family 27 subfamily A member 1; *BACS*, Bile acid CoA ligase; *BAAT*, bile acid-CoA:amino acid N-acyltransferase; *NTCP*, Na+-taurocholate co-transporting polypeptide; *OATP*, organic-anion-transporting polypeptides; *BSEP*, bile salt export pump; *MDR2*, multidrug resistance-associated protein 2; *MDR3*, multidrug resistance-associated protein 3; *OSTA*, organic solute transporter α; *OSTB*, organic solute transporter β; *SREBP1*, sterol regulatory element-binding transcription factor 1; *ACC*, acetyl-coenzyme A carboxylase; *PPARG*, peroxisome proliferator-activated receptor gamma; *G6PC*, glucose-6-phosphatase; *PEPCK*, phosphoenolpyruvate carboxykinase.

qRT-PCR analysis of hepatic tissues revealed that *SHP* mRNA levels were significantly decreased in patients with MASH compared with those without (Fig. 4). The mRNA levels of *CYP7A1*, a key BA-synthesis enzyme, were significantly increased in the MASH group. Additionally, genes associated with BA metabolism, including alternative BA synthetic enzymes (*CYP7B1* and *CYP27A1*), BA conjugating enzyme (*BAAT*), BA importers (*NTCP*, *OATP1B1*, and *OATP1B3*), and BA exporters (*BSEP*, *MDR2*, and *MDR3*), were also upregulated, whereas the mRNA levels of alternative BA spillover exporters (*OSTA* and *OSTB*) showed no significant differences, indicating that the gene expression profile favors BA accumulation in patients with MASH.

These observations prompted us to examine the conventional inhibitory role of SHP by assessing the correlations among SHP nuclear localization, BA metabolism-associated genes, and serum BA levels. The results showed that SHP nuclear localization was positively correlated with *CYP7A1* mRNA levels, without a significant association with most serum BA categories (Table S5). Therefore, although patients with MASH exhibit a hepatic mRNA profile that favors cholestasis, BA accumulation may not be directly associated with hepatic SHP nuclear localization and is irrelevant to its expected role in suppressing BA synthesis.

We analyzed the expression of key gluconeogenic genes, phosphoenolpyruvate carboxykinase (*PEPCK)* and glucose-6-phosphatase (*G6PC*), and observed that they were upregulated in participants with MASH, especially for *G6PC.* Further stratification by fibrosis stage revealed that the expression levels of both genes decreased with increasing disease severity (Fig. S5).

### PKCζ activation mediates SHP nuclear localization induced by toxic fatty acid overload and inflammatory stimulation

**Fig. 5:**
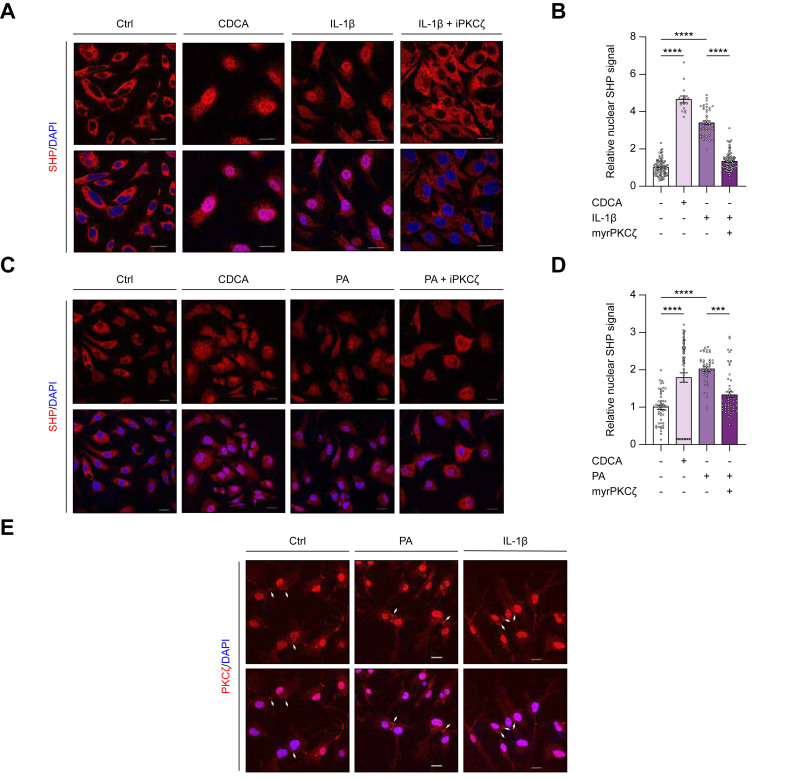
Nuclear SHP translocation is triggered by lipotoxicity and inflammation and mediated by PKCζ activation. (A–D) In HepG2 cells, exposure to IL-1β (3rd panel of A and B) or palmitic acid (PA, 3rd panel of C and D), similar to chenodeoxycholic acid (CDCA, 2nd panel of A–D), triggered SHP nuclear translocation. PKCζ inhibition by myrestaloid PKCζ pseudosubstrate (iPKCζ, 4th panel of A–D) reduced SHP nuclear translocation. (E): PA and IL-1 β enhance PKCζ signal intensity (white arrows) at cell–cell junctions. Scale bar = 20 μm (∗*p* <0·05, ∗∗*p* <0·01, ∗∗∗*p* <0·001, Kruskal–Wallis test). CDCA, chenodeoxycholic acid; PA, palmitic acid; PKCζ, protein kinase C zeta; SHP, small heterodimer partner; myr-, myristoylated.

To investigate the mechanisms underlying the elevated SHP nuclear localization in patients with MASH, we conducted *in vitro* experiments using HepG2 cells and primary human hepatocytes (PHHs). Given the observed association between SHP nuclear localization, hepatic steatosis, and inflammation in MASH, we examined SHP nuclear localization following exposure to excess palmitic acid (PA) and IL-1β, alongside the canonical SHP inducer, CDCA. The results demonstrated that both PA and IL-1β significantly promoted SHP nuclear translocation (Fig. 5A, C, left three panels, and Fig. S6A). Quantitative analysis of SHP nuclear signal intensity further confirmed the elevation of SHP nuclear translocation in response to IL-1β, PA, and CDCA, with IL-1β treatment eliciting the most pronounced effect (Fig. 5B and D, and Fig. S6B).

Given that PKCζ mediates SHP nuclear translocation, we further explored its role in PA- and IL-1β-induced SHP nuclear localization. The PKCζ signal was presented as a linear pattern along the cell surface at sites of cell–cell contact. After treatment with PA and IL-1β, the signal intensity of PKCζ in the cell–cell junctional area was more pronounced (Fig. 5E). Using an N-myristoylated pseudosubstrate inhibitor to block PKCζ activity, we observed that PKCζ inhibition effectively attenuated SHP nuclear translocation triggered by PA and IL-1β (Fig. 5A and C, right panel, and Fig. S6). These findings suggest that PA and IL-1β promote SHP nuclear localization in hepatocytes by activating PKCζ.

**Fig. 6:**
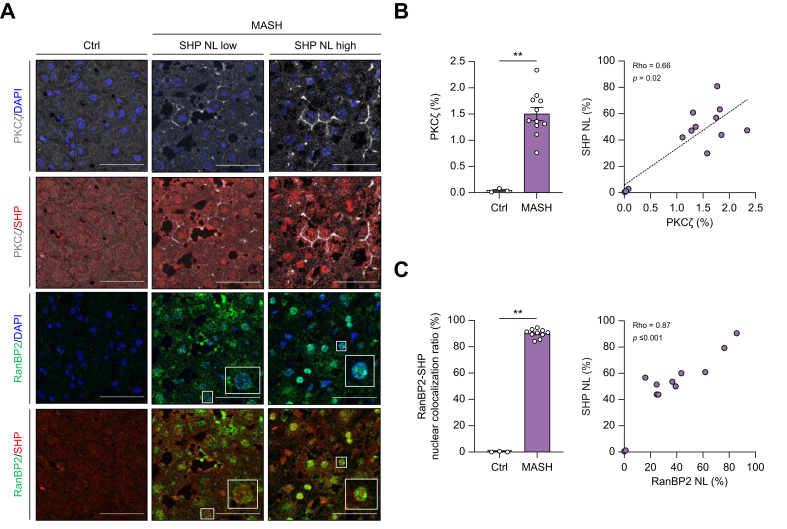
Immunofluorescent co-staining of PKCζ, RanBP2, and SHP in human hepatic tissue. (A) In patients with MASH, hepatic PKCζ (gray) signal intensity is significantly increased and exhibited linear and hexagonal shapes (upper two panels). The RanBP2 (green) signal intensity was highly co-localized with SHP (red) in the nuclear/perinuclear area of hepatocytes (lower two panels). (B and C) Quantification of the hepatic signal intensity of PKCζ (B) and RanBP2 (C), as well as their correlation with the nuclear ratio of SHP. Scale bar = 50 μm. (∗∗*p* <0.01, Mann–Whitney *U* test, Rho: Spearman’s correlation coefficient). MASH, metabolic dysfunction-associated steatohepatitis; PKCζ, protein kinase C zeta; RanBP2, RAN binding protein 2; SHP, small heterodimer partner; SHP NL, nuclear SHP.

To extend these *in vitro* findings to a clinical context, we performed co-immunostaining of SHP with PKCζ and nucleoporin RanBP2 in liver tissue samples. RanBP2 is also known as a key factor that induces SHP nuclear translocation upon BA stimulation.^20^ The results revealed that in patients with MASH demonstrated a linear to hexagonal perimembranous distribution pattern indicative of PKCζ activation (Fig. 6A, upper two panels), along with a significant increase in PKCζ signal intensity (Fig. 6B, left panel). Furthermore, hepatic PKCζ signal intensity strongly correlated with SHP nuclear localization (Fig. 6B, right panel). Similarly, RanBP2 expression was significantly elevated in patients with MASH, particularly in the nuclear and perinuclear regions (Fig. 6A, lower two panels, and Fig. 6C, left panel). Notably, RanBP2 was most prominently expressed in areas with nuclear SHP localization, suggesting a high degree of nuclear co-localization between SHP and RanBP2 (Fig. 6A, lower two panels, and Fig. 6C, right panel).

Both PKCζ activation and RanBP2-SHP nuclear co-localization were evaluated in relation to the serum markers of hepatic inflammation and metabolic dysfunction. PKCζ signal intensity positively correlated with serum γ-GT levels, whereas RanBP2-SHP nuclear co-localization was associated with ALT and AST levels. Additionally, PKCζ signal intensity correlated with metabolic biomarkers, including fasting serum TGs, glucose, and HbA1c levels, whereas RanBP2-SHP nuclear co-localization was strongly associated with BMI. Furthermore, PKCζ signal intensity showed a robust positive correlation with the degree of hepatic steatosis. In contrast, PKCζ intensity and RanBP2-SHP nuclear co-localization were not associated with serum BA levels (Table 2).

**Table 2:** The correlations of hepatic PKCζ signal intensity and RanBP2-SHP nuclear co-localization ratio (RanBP2-SHP coNL) with serum hepatitis markers, parameters related to metabolic dysfunction, degree of hepatic tissue steatosis, and serum bile acid levels.

	PKCζ signal intensity	RanBP2-SHP coNL
	Rho (*p* value)	Rho (*p* value)
ALT	0.48 (0.098)	0.73 (0.004)∗∗
AST	0.45 (0.128)	0.56 (0.047)∗
γ-GT	0.73 (0.005)∗∗	0.53 (0.06)
ALP	0.28 (0.348)	0.28 (0.37)
Ferritin	0.18 (0.627)	0.48 (0.11)
LSM	-0.21 (0.556)	-0.02 (0.96)
CAP	-0.11 (0.76)	0.41 (0.24)
ARFI	0.02 (0.96)	-0.09 (0.82)
BMI	0.38 (0.194)	0.81 (<0.001)∗∗∗
TG‡	0.67 (0.023)∗	0.59 (0.09)
Cholesterol	-0.12 (0.707)	0.53 (0.08)
LDL	-0.18 (0.627)	0.24 (0.48)
HDL	0.27 (0.444)	0.54 (0.08)
HOMA-IR‡	-0.29 (0.535)	0.06 (0.89)
Insulin‡	-0.46 (0.294)	0.04 (0.93)
HbA1C‡	0.85 (0.016)∗	0.63 (0.25)
Fasting glucose ‡	0.67 (0.033)∗	0.26 (0.50)
Steatosis (%)†	0.90 (0.00003)∗∗∗	0.60 (0.03)∗
Total BA	0.46 (0.117)	0.52 (0.08)
Conjugated BA	0.41 (0.162)	0.21 (0.51)
Unconjugated BA	0.34 (0.255)	0.46 (0.13)
Primary BA	0.47 (0.103)	0.53 (0.08)
Secondary BA	0.24 (0.426)	0.33 (0.30)

Rho: Spearman’s correlation coefficient (∗*p* <0.05, ∗∗*p* <0.01, ∗∗∗*p* <0.001).

γ-GT, gamma-glutamyl transferase; ALP, alkaline phosphatase; ALT, alanine transaminase; ARFI, acoustic radiation force impulse; AST, aspartate aminotransferase; BA, bile acid; CAP, controlled attenuation parameter; HbA1C, glycosylated hemoglobin A1C; HOMA-IR, Homeostatic Model of Assessment Insulin Resistance; LSM, liver stiffness measurement; PKCζ, protein kinase C zeta; RanBP2, RAN binding protein 2; SHP, small heterodimer partner.

† Semiquantitative degree by pathologist.

‡ Only enrolled those not taking medications for type II diabetes mellitus or dyslipidemia.

These findings suggest that PKCζ activation and nuclear/perinuclear localization of RanBP2 play critical roles in SHP nuclear translocation and are closely associated with hepatic inflammation, steatosis, and metabolic dysfunction in patients with MASH.

### In MASH-related conditions, SHP silencing enhances innate immune mediators and stimulates a cholestatic gene signature, whereas overexpressing SHP attenuates the inflammatory effects

To address the effects of *SHP* silencing on inflammation and immunity in MASH-related conditions, we searched the Gene Expression Omnibus (GEO) database for genome-wide, high-throughput datasets. Two datasets (accession numbers GSE38013^39^ and GSE133566^15^) featuring liver-specific *SHP* knockdown in MASH mouse models were identified. Functional annotation analysis using the Database for Annotation, Visualization, and Integrated Discovery (DAVID)^40^^,^^41^ revealed that *SHP* knockdown significantly altered gene clusters primarily associated with the immune system, particularly innate immune responses (Fig. S7).

**Fig. 7:**
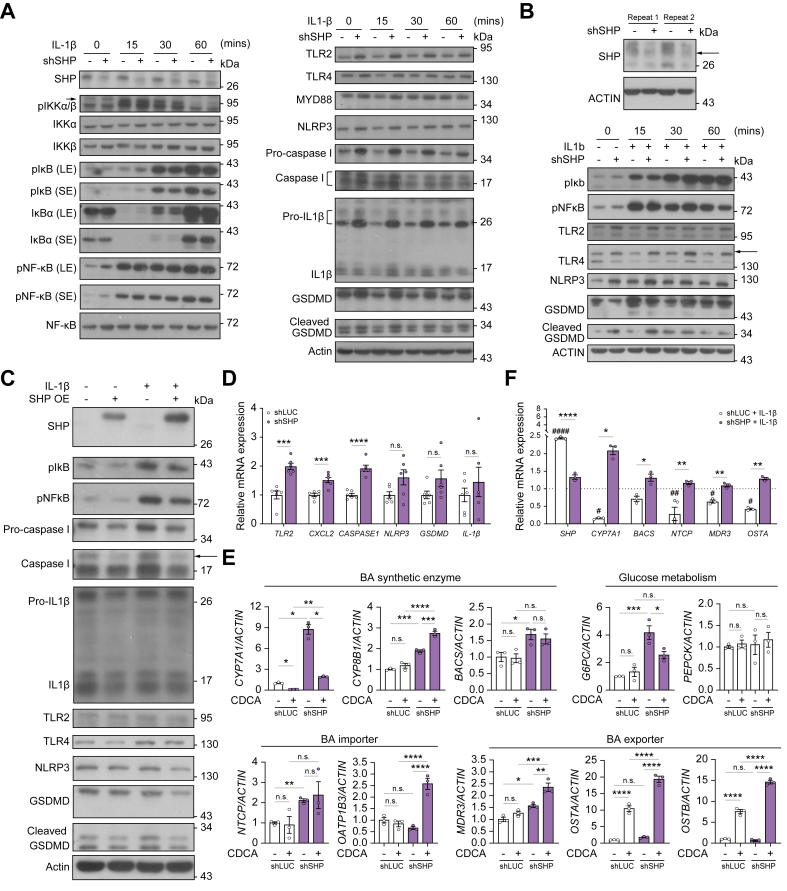
SHP negatively controls innate immune response and prevents cholestasis. (A and B) Western blot of signaling proteins involved in the activation of NF-κB (left panel) and innate immunity (right panel) in HepG2 cells (A) and in primary human hepatocytes (B) with *SHP* knockdown. (C) *SHP* overexpression in HepG2 cells attenuates innate immune response. (D–F) *SHP* knockdown in HepG2 cells increased basal mRNA levels of innate immune mediators (D) and BA-related genes (E). (F) IL-1β treatment attenuates BA-related genes (white bar)*,* whereas *SHP* knockdown enhanced their expression (gray bar). Dot reference line: shLUC cells without IL-1β treatment. (D and E: ∗*p* <0.05, ∗∗*p* <0.01, ∗∗∗*p* <0.001, Mann–Whitney *U* test for two groups and Kruskal–Wallis test for three or more groups, (F) Comparison of shLUC cells treated with or without IL-1β, ^#^*p* <0.05, ^##^*p* <0.01. shLUC or shSHP cells treated with IL-1β, ∗*p* <0.05, ∗∗*p* <0.01, ∗∗∗*p* <0.001). BA, bile acid; SHP, small heterodimer partner; BAAT, bile acid-CoA:amino acid N-acyltransferase; BSEP, bile salt export pump; CYP7A1, cytochrome P450 family 7 subfamily A member 1; CYP7B1, cytochrome P450 family 7 subfamily B member 1; GSDMD, gasdermin D; IKK, IκB kinase; IL-1β, interleukin-1β; MDR2, multidrug resistance-associated protein 2; MDR3, multidrug resistance-associated protein 3; MYD88, myeloid differentiation primary response 88; NF-κB, nuclear factor kappa B; NLRP3, NLR family pyrin domain containing 3; NTCP, Na+-taurocholate co-transporting polypeptide; OATP, organic-anion-transporting polypeptides; OSTA, organic solute transporter α; OSTB, organic solute transporter β; pIkB, phosphorylated IkB; pNF-κB, phosphorylated NF-κB; TLR2, Toll-like receptor 2; TLR4, Toll-like receptor 4.

To further explore this finding, we examined the effects of *SHP* knockdown and overexpression on IL-1β-induced NF-κB activation and innate immunity in HepG2 cells. The results showed that *SHP* knockdown increased phosphorylated NF-κB (pNF-κB) levels in the basal state and increased phosphorylation of IkB (pIkB) after IL-1β treatment for 15 min but did not affect IL-1β-induced phosphorylation of IKK or the degradation of IκB within 60 min (Fig. 7A, left panel). Next, we analyzed the protein levels of innate immune receptors, mediators, and NLRP3 inflammasome components. *SHP* knockdown led to increased basal protein levels of Toll-like receptors (TLR2 and TLR4), myeloid differentiation primary response 88 (MYD88), NLR family pyrin domain containing 3 (NLRP3), pro-caspase 1, pro-IL-1β, and gasdermin D (GSDMD). Additionally, these factors exhibited transient upregulation following IL-1β treatment, peaking at 15 min (Fig. 7A, right panel). Consistently, *SHP* knockdown following IL-1β stimulation for long term increased the levels of pNF-κB, TLR2, pro-caspase-1, and GSDMD regardless of IL-1β treatment (Fig. S8).

Experiments using PHHs showed results comparable to those observed for HepG2 cells. *SHP* knockdown increased basal levels of NF-κB signaling molecules, including pIκb and pNF-κB, and innate immune mediators, including TLR2, TLR4, NLRP3, and cleaved GSDMD. IL-1β treatment increased the protein levels of TLR2, TLR4, NLRP3, and cleaved GSDMD in *SHP* knockdown cells (Fig. 7B).

*SHP* overexpression significantly attenuated the levels of pNF-κB, pIκB, NLRP3, and GSDMD following IL-1β stimulation, and reduced the basal protein levels of caspase-1, pro-IL-1β, and mature IL-1β (Fig. 7C). The overexpression of *SHP* tended to decrease the basal levels of pNF-κB, pIκB, pro-caspase-1, and TLR-4 (Fig. 7C and Fig. S9). The mRNA levels of innate immune mediators, including *TLR2*, *CXCL2*, and *CASPASE1*, were increased after *SHP* knockdown. *NLRP3*, *GSDMD*, and *IL1B* mRNA levels tended to increase following *SHP* knockdown (Fig. 7D). However, the effect of *SHP* overexpression on inflammatory gene expression was not as robust as that of *SHP* knockdown, as reflected by the lack of significant changes in the tested mRNA levels (Fig. S10). These findings suggest that SHP plays a negative regulatory role in innate immune responses in MASH-related conditions.

To investigate the role of SHP in regulating BA homeostasis in MASH, we examined the effects of *SHP* silencing on the expression of BA metabolic genes following CDCA treatment. *SHP* knockdown led to the upregulation of key BA metabolic genes, including those involved in BA synthesis (*CYP7A1, CYP8B1,* and *BACS*) and transport (*NTCP* and *MDR3*). The most prominent increase was observed in *CYP7A1* expression, as it was upregulated by approximately eightfold compared with that in the shLUC control cells (Fig. 7E). As expected, CDCA treatment effectively suppressed *CYP7A1* expression in shLUC control cells; however, this suppression was significantly attenuated in shSHP cells (*CYP7A1* reduction: 91.4% *vs.* 78.0%, *p* = 0.004; Fig. 7E). Additionally, SHP silencing enhanced the CDCA-induced upregulation of several BA metabolic genes, including *CYP8B1, OATP1B3, MDR3, OSTA,* and *OSTB* (Fig. 7E). *SHP* knockdown also upregulated the gluconeogenic gene, *G6PC*, which was suppressed by CDCA treatment (Fig. 7E). These findings suggest that SHP plays a crucial role in maintaining a gene expression profile that mitigates cholestasis and gluconeogenesis.

As IL-1β can induce SHP nuclear translocation, we further examined its effects on BA metabolic genes. IL-1β treatment significantly increased *SHP* mRNA levels while suppressing multiple BA metabolic genes, including *CYP7A1*, *NTCP*, and *MDR3* and *OSTA*, in shLUC control cells (Fig. 7F, white bars). SHP silencing attenuated the inhibitory effects of IL-1β on *CYP7A1, NTCP, MDR3,* and *OSTA*, while simultaneously upregulating *BACS*, *G6PC*, and acetyl-coenzyme A carboxylase (*ACC*) (Fig. 7F, gray bars). These gene expression patterns promoted a cholestatic profile that closely resembled the molecular signature observed in patients with MASH, highlighting the importance of SHP in preventing BA accumulation.

### SHP knockdown promotes lipid accumulation and upregulates genes related to *de novo* lipogenesis

**Fig. 8:**
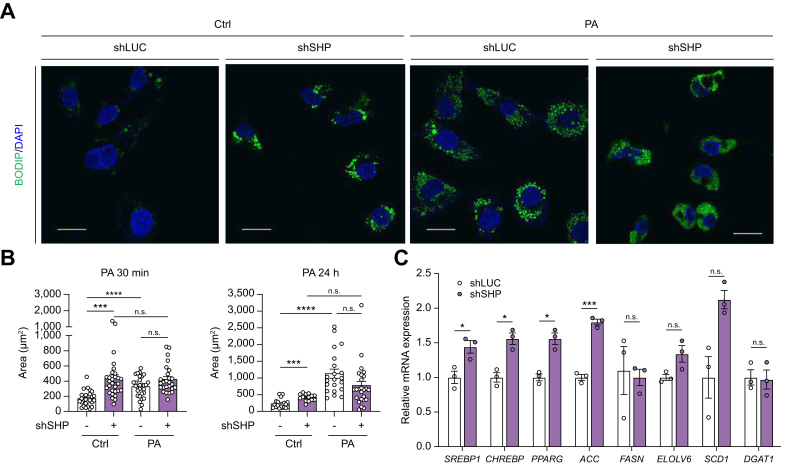
*SHP* knockdown induces hepatocellular steatosis *in vitro*. (A) Immunofluorescent staining of lipid droplets using BODYPI staining (green) in HepG2 cells with wild-type (shLUC) or *SHP* knockdown (sh*SHP*), treated with PA for 24 h. (B) Quantification of lipid droplets in shLUC and sh*SHP* HepG2 cells following PA treatment for 30 min (left panel) or 24 h (right panel). (C) Quantification of mRNA levels in genes related to *de novo* lipogenesis under basal conditions in HepG2 cells with wild-type or *SHP* knockdown. Scale bar = 50 μm ∗*p* <0.05, ∗∗*p* <0.01, ∗∗∗*p* <0.001, Mann–Whitney *U* test (for two groups) and Kruskal–Wallis test (for ≥3 groups). PA, palmitic acid; SHP, small heterodimer partner.

We investigated the effects of *SHP* knockdown on lipid accumulation and lipogenic gene expression over time. *SHP* knockdown significantly increased lipid accumulation under basal conditions over time (Fig. 8A and B). In control cells, PA exposure for 30 min (short term) and 24 h (long term) markedly increased lipid accumulation, reaching levels comparable with those observed in untreated *SHP* knockdown cells. In contrast, PA exposure did not further enhance lipid accumulation in *SHP* knockdown cells. Consistently, SHP knockdown led to increased basal mRNA expression of key genes involved in *de novo* lipogenesis, including sterol regulatory element-binding transcription factor 1 (*SREBP1*), *CHREBP*, peroxisome proliferator-activated receptor gamma (*PPARG*), and *ACC* (Fig. 8C).

## Discussion

In this study, an elevated nuclear SHP ratio emerged as a distinctive hallmark of tissue pathology in patients with MASH. We identified a strong association between nuclear SHP localization, hepatic inflammation, and steatosis, linking them to atypical PKCζ activation and perinuclear/nuclear aggregation of nucleoporin RanBP2 in liver tissue. *In vitro* experiments showed that increased hepatocellular steatosis and innate immune mediators after *SHP* knockdown and decreased innate immune responses following *SHP* overexpression indicate its role in suppressing hepatic steatosis and inflammation. However, despite the increased nuclear SHP localization in patients with MASH, serum BA levels were significantly elevated. Moreover, nuclear SHP localization positively correlated with BA synthesis-related genes in patients with MASH, rather than showing the expected negative correlation. This suggests that other MASH-dependent factors contribute to BA upregulation in steatohepatitis. *In vitro* SHP knockdown induced a cholestatic gene profile mirroring the changes observed in patients with MASH. Collectively, our study highlights the pivotal role of SHP in alleviating hepatic inflammation, steatosis, and cholestasis in patients with MASH, whereas its suppressive function with BA synthesis appears to be diminished.

Studies on hepatic SHP distribution in humans and its disease associations remain limited. Zou *et al.*^18^ reported that SHP protein levels decreased in the livers of patients with MASH but remained unchanged in patients with simple steatosis compared with those of control individuals. However, this study did not assess the subcellular localization of *SHP* in liver tissue. Wilczek *et al.*^42^ showed that SHP is predominantly localized in the cytoplasm rather than the nucleus in healthy human liver. This finding is consistent with the results obtained in our study.

SHP interacts with NF-κB to mitigate inflammatory responses.^16^^,^^17^ Our experiments demonstrated that *SHP* knockdown enhanced basal NF-κB phosphorylation and increased IκB phosphorylation upon IL-1β treatment, whereas *SHP* overexpression had the opposite effect. These findings suggest that SHP suppresses NF-κB under steady-state conditions and prevents its activation during inflammatory stimuli. SHP also regulates innate immune responses, as Yang *et al.*^16^ showed that SHP interacts and negatively regulates the NLRP3 inflammasome. Our study demonstrated that *SHP* knockdown increased basal levels of pro-caspase-1, pro-IL-1β, NLRP3, and GSDMD, priming the NLRP3 signaling pathway and facilitating inflammasome activation, and increased TLR2, TLR4, implicating SHP in suppressing innate immune activation. *SHP* overexpression attenuated innate immune responses. GEO database analysis revealed that hepatic *SHP* knockdown in the MASH animal model significantly increased MASH severity and altered genes related to innate immunity, whereas *SHP* overexpression attenuated hepatic injury,^15^^,^^18^ supporting observations of the present study. This upregulation occurred at baseline and persisted throughout treatment, suggesting that SHP restrains resident immune proteins, preventing and mitigating excessive immune activation.

Nuclear translocation of SHP relies on PKCζ activity and post-translational modification by RanBP2. Seok *et al.*^19^ and Kim *et al.*^20^ identified PKCζ as a key regulator of SHP activation, priming it for nuclear translocation through phosphorylation. RanBP2-mediated SUMOylation is critical for SHP nuclear localization.^19^^,^^20^ In HepG2 cells, inhibition of PKCζ or knockdown of RanBP2 significantly impaired SHP nuclear repressive function, including its suppression of BA synthesis. Meanwhile, studies suggesting that PKCζ-mediated phosphorylation occurs upstream of RanBP2-mediated SUMOylation.^19^^,^^20^ Consistent with previous studies, our immunofluorescent staining of human liver tissues demonstrated that PKCζ activation and RanBP2 aggregation occurred in the perinuclear/nuclear regions of hepatocytes, where they were highly co-localized with nuclear SHP in patients with MASH. Furthermore, our *in vitro* findings reinforce the critical role of PKCζ in mediating SHP nuclear translocation. First, we demonstrated that both IL-1β and PA induced SHP nuclear translocation and significantly enhanced signal intensity of PKCζ activation at cell–cell junctions. Second, we showed that PKCζ inhibition reduced SHP nuclear translocation in response to CDCA stimulation and under conditions involving toxic lipid (PA) and inflammatory cytokine (IL-1β) treatment. These results suggest that pathological conditions of steatohepatitis may trigger PKCζ-mediated SHP nuclear translocation.

Several studies have demonstrated that hepatic atypical PKC activation is associated with lipid droplet accumulation and insulin resistance in patients with MASH and cultured hepatocytes.^24^^,^^25^^,^[43], [44], [45] PKCζ activation is typically indicated by its translocation from the cytosol to the plasma membrane.^46^ Our study revealed a distinct linear and hexagonal distribution of PKCζ in hepatic tissues, suggesting significant PKCζ activation in patients with MASH. This finding was further supported by the positive correlation between PKCζ signal intensity in liver tissues and the severity of hepatic steatosis. However, PKCζ signal intensity showed a significant positive correlation with serum parameters of hepatitis, suggesting a potential link between hepatic inflammation and PKCζ activation. This was further supported by our *in vitro* study, which demonstrated that both toxic fatty acids and inflammatory cytokines enhanced PKCζ signal intensity at the cell membrane.

Our study revealed significant nuclear/perinuclear co-localization of RanBP2 with SHP, which was strongly associated with serum hepatitis markers and the degree of hepatic steatosis in patients with MASH. Given that RanBP2 has been implicated in lipid homeostasis under oxidative stress,^47^ it is possible that RanBP2 is also involved in MASH initiation and progression. The causal relationship between PKCζ activation, RanBP2, and MASH warrants further investigation.

Increased serum BA levels in patients with MASH have been previously reported.^11^^,^^13^^,^[48], [49], [50] In line with these studies, our data also demonstrated significant elevations in BA levels in patients with MASH, although no specific BA was directly linked to disease severity. The underlying mechanisms remain unclear. Previous studies have reported elevated hepatic *CYP7A1* mRNA levels in patients with MASH.[11], [12], [13] In our study, the substantial upregulation of *CYP7A1* and other BA-related genes, accompanied by a modest increase in BA exporter genes, suggests a relatively cholestatic profile in MASH, which may partially explain the elevated serum BA levels and dysregulation of BA homeostasis in these patients.

Although *SHP* overexpression protects against steatohepatitis in murine models, our study and a previous report^11^ showed that *SHP* expression was downregulated in MASH. This downregulation appears to be driven by inflammatory and lipotoxic signals, such as palmitic acid and LPS, which activate the JNK pathway and lead to c-Jun-mediated repression of *Shp* transcription.^18^ Resulting decrease in SHP relieves its inhibitory control over NF-κB signaling, particularly chemokine CCL2 expression, promoting macrophage infiltration and hepatic inflammation. Thus, *SHP* mRNA downregulation in MASH is not paradoxical but is a key component of disease progression from steatosis to steatohepatitis. SHP also exhibits a negative regulatory mechanism by inhibiting its transcription through interactions with the chromatin-remodeling complex (SWItch/Sucrose Non-Fermentable, SWI/SNF) in the SHP promoter region.^51^^,^^52^ It is likely that SHP attenuates its transcription and forms a negative feedback loop.

Our study and a previous report^53^ showed that gluconeogenic genes were upregulated in the MASH cohort and decreased with increasing disease severity (Fig. S5). Recent evidence suggests that PEPCK plays a protective role in ameliorating hepatic steatosis, whereas hepatic PEPCK deficiency promotes inflammation and fibrogenesis in MAFLD mouse models.^53^ We propose that gluconeogenic genes exert protective effects in the early stages of MAFLD or MASH. Their upregulation may represent an adaptive response to counteract disease progression, which diminishes as fibrosis advances.

Our findings are constrained by the absence of direct biochemical proof for protein–protein binding between SHP and related proteins in human MASH tissues. Although immunofluorescence staining clearly demonstrated enhanced nuclear localization of SHP in patient samples, this approach cannot establish physical interactions between proteins. To further support the protein–protein interactions, we conducted the integrating network analyses from STRING-DB (https://string-db.org/). According to STING-DB, only one study has reported that SHP interacts with NF-κB p65 to attenuate inflammatory responses in both a mouse macrophage cell model and a murine knockout model.^18^ To the best of our knowledge, the interaction of SHP with inflammatory signaling has not been addressed in human hepatocytes. The inflammatory signaling cascades may differ substantially between human hepatocytes and murine models.[54], [55], [56] Further studies will be required to investigate the SHP interactome in human tissues and cells.

Although a substantial proportion of patients in our cohort were receiving statins or antidiabetic therapies, subgroup analyses indicated no significant modification of the main results. Nevertheless, these agents are known to directly or indirectly modulate IL-1β signaling, in addition to their effects on lipid metabolism and SHP-related pathways. Therefore, we cannot fully exclude the possibility that IL-1β–driven inflammatory signaling may have been influenced by these medications in our findings.

In conclusion, increased hepatic SHP nuclear localization is associated with key pathological characteristics, including hepatic inflammation, steatosis, and metabolic syndrome, in patients with MASH; however, it does not emerge as a traditionally recognized suppressive factor that is negatively correlated with BA synthesis. Mechanistically, SHP nuclear translocation is most likely triggered by inflammatory and lipotoxic stimuli and is dependent on PKCζ activity. Consistently, PKCζ activation and RanBP2 co-localization with nuclear SHP were prominent in the liver tissue of patients with MASH in this study. *In vitro*, SHP knockdown enhances innate immune components and hepatocellular steatosis, and recapitulates a cholestatic gene signature that has been observed in patients with MASH, suggesting that increased SHP nuclear translocation may serve to mitigate steatosis and innate immune responses, as well as prevent the cholestatic phenotype. Collectively, our study identified elevated SHP nuclear localization as a novel hallmark of MASH, which might represent a compensatory response aimed at anti-inflammation and anticholestasis in the face of the inflammation and lipotoxicity of MASH.

## Abbreviations

γ-GT, gamma-glutamyl transferase; ACC, acetyl-coenzyme A carboxylase; AI, artificial intelligence; ALP, alkaline phosphatase; ALT, alanine transaminase; AST, aspartate aminotransferase; BA, bile aid; BACS, Bile acid CoA ligase; BAAT, bile acid-CoA:amino acid N-acyltransferase; BSEP, bile salt export pump; CA, cholic acid; CDCA, chenodeoxycholic acid; CYP27A1, cytochrome P450 family 27 subfamily A member 1; CYP7A1, cytochrome P450 family 7 subfamily A member 1; CYP7B1, cytochrome P450 family 7 subfamily B member 1; DAVID, Database for Annotation, Visualization, and Integrated Discovery; G6PC, glucose-6-phosphatase; GCDCA, glyco-CDCA; GEO, Gene Expression Omnibus; GSDMD, gasdermin D; HbA1C, glycosylated hemoglobin A1C; HOMA-IR, Homeostatic Model of Assessment Insulin Resistance; IKK, IκB kinase; IL-1β, interleukin-1β; LSM, liver stiffness measurement; MASH, metabolic dysfunction-associated steatohepatitis; MASLD, metabolic dysfunction-associated steatotic liver disease; MDR2, multidrug resistance-associated protein 2; MDR3, multidrug resistance-associated protein 3; MYD88, myeloid differentiation primary response 88; NAFLD, non-alcoholic fatty liver disease; NAS, NAFLD Activity Score; NASH-CRN, NASH-Clinical Research Network; NCKUH, National Cheng Kung University Hospital; NF-κB, nuclear factor kappa B; NLRP3, NLR family pyrin domain containing 3; NTCP, Na+-taurocholate co-transporting polypeptide; OATP, organic-anion-transporting polypeptides; OSTA, organic solute transporter α; OSTB, organic solute transporter β; PA, palmitic acid; PEPCK, phosphoenolpyruvate carboxykinase; PHHs, primary human hepatocytes; pIkB, phosphorylated IkB; PKC, protein kinase C; PKCζ, protein kinase C zeta; pNF-κB, phosphorylated NF-κB; PPARG, peroxisome proliferator-activated receptor gamma; qRT-PCR, quantitative reverse transcriptase-PCR; RanBP2, RAN binding protein 2; SHP, small heterodimer partner; SREBP1, sterol regulatory element-binding transcription factor 1; TCDCA, tauro-CDCA; TG, triglyceride; TLR2, Toll-like receptor 2; TLR4, Toll-like receptor 4.

## Financial support

This work was supported by grants from the 10.13039/100020595National Science and Technology Council, Taiwan (110-2314-B-006-018, 112-2314-B-006-070, 113-2314-B-006-060, 110-2320-B-006-017-MY3, 111-2320-B-006-022-MY3, and 113-2320-B-006-039-MY3), 10.13039/501100004844National Cheng Kung University Hospital, Taiwan (NCKUH-10603024, NCKUH-10804029, NCKUH-10902057, NCKUH-11106004, NCKUH-11204002, and NCKUH-11404018), and Hsu‑Yuan Education Foundation.

## Authors’ contributions

Drafting and manuscript composition: S-CC. Original concept and study design: S-CC, C-YC, Y-ST. Interpretation of human pathology: H-WT. BA data analysis: S-CS. Collection of living donor individuals: Y-JL. Recruitment of MASH individuals: S-CC, H-CC, Y-CC, P-NC. Guidance of culturing primary hepatocytes: K-CY. Data curation and methodology: M-SW, Y-HL, M-JZ, S-CC. Draft writing and editing: S-CC.

## Data availability

The datasets generated or analyzed in the presented study are available from the corresponding author on reasonable request.

## Conflicts of interest

The authors declare no conflicts of interest that pertain to this work.

Please refer to the accompanying ICMJE disclosure forms for further details.
