# Supplementary material for: Enhanced nuclear localization of small heterodimer partner in metabolic dysfunction-associated steatohepatitis
Source: JHEP Rep. 2025 Oct 4;8(1):101616. doi: 10.1016/j.jhepr.2025.101616 (PMC12721038; doi:10.1016/j.jhepr.2025.101616)
Supplement: Multimedia component 1 [file mmc1.pdf]

Title:

Distinct role of nuclear-localized small heterodimer partner in metabolic dysfunction-associated steatohepatitis

Shih-Chieh Chien[2,1], Chiung-Yu Chen[2], Hung-Wen Tsai[3], Yih-Jyh Lin[4], Shu-Chu Shiesh[5], Pin-Nan Cheng[2], Hung-Chih Chiu[2], Yen-Cheng Chiu[2], Ya-Han Lin[1], Min-Shan Wu[1], Mei-Juan Zheng[1], Kung-Chia Young[5], Yau-Sheng Tsai[1,6]

Table of contents

Supplementary methods……………………………………………………………………2

Supplementary figures……………………………………………………………………6

Supplementary tables…………………………………………………………………...15

Material and method for human tissue immunofluorescent stating and quantification

Immunofluorescent staining: The primary antibodies used in the study included those targeting SHP (GTX54598, GeneTex), NF-κB p65 subunit (sc-372, Santa Cruz), PKCζ (sc-17781, Santa Cruz), and RanBP2 (sc-74518). Paraffin-embedded hepatic sections were deparaffinized, blocked, and subsequently incubated overnight with the abovementioned primary antibodies in 3% bovine serum albumin at 4℃. These sections were further incubated with horseradish peroxidase-conjugated secondary antibodies using the Opal™ 4-Color Automation Immunohistochemistry Kit (NEL820001KT). Micrographs were acquired using a confocal laser-scanning microscope (FV3000, Olympus).

Tissue staining quantification: All immunofluorescent-stained slides of liver samples were viewed using TissueFAXS plus fluorescent-activated cell sorter-like tissue cytometry (TissueGnostics). The nuclear localization of SHP and NF-κB was confirmed by the co-localization of their respective antibody-derived signal with that of 4′,6-diamidino-2-phenylindole (DAPI). The ratios of nuclear SHP and NF-κB, expressed as a percentage per area of interest, were quantified through colorimetric analysis using TissueQuest 7.1 software (TissueGnostics).

Material and method for *in vitro* experiments

Cell culture and treatment: HepG2 cell lines (ATCC HB-8065) were cultured in high glucose DMEM cell culture medium containing 10% fetal bovine serum (FBS). Primary human hepatocytes (PHHs, SC-0413) were cultured in hepatocyte medium (HM, No. 5201, ScienCell) containing 5% FBS and 1% hepatocyte growth supplement (HGS, No. 5252, ScienCell) and 1% antibiotic solution (P/S, No. 0503, ScienCell). All of the cell lines were grown in 5% CO_2 in a humidified incubator maintained at 37℃. For the treatment, cells with the density of $5*10^4$ cells/well were incubated in maintenance medium in 12-well plates overnight, and replaced with serum-free medium for 6 hours, subsequently stimulated with 50 μM of chenodeoxycholic acid (CDCA) (C2861, Sigma-Aldrich), 200 μM of palmitic acid (PA, No. 29558, Cayman), 5 ng/mL (PHHs) or 10 ng/mL (HepG2) of IL-1β (No. 200-01B; Peprotech) with or without 5 μM of myristoylated PKCζ pseudosubstrate (myr-PKCζ pseudosubstrate, ab120993; Abcam) for 30 minutes.

Extraction and purification of plasmids: Various plasmids were transformed into E. coli DH5α competent cells and cultured overnight in LB or TB medium supplemented with the appropriate antibiotics at 37 °C with shaking at 225 rpm. The bacterial cells were then harvested, and plasmids were extracted and purified using the Biokit Plasmid Miniprep Kit (Bio-P300), following the manufacturer's instructions.

Knockdown of SHP: HepG2 cells were cultured in maintenance medium overnight, infected with lentivirus containing NR0B2-specific small hairpin RNAs (shRNA, CACATTGGACTTCCTTGGTTT) at a multiplicity of infection (MOI) of ~$5*10^6$ TU/mL for 48 hours, and recovered in maintenance medium for 24 hours. Finally,

cells were selected in a maintenance medium with puromycin (5 µg/mL) for 7 to 10 days.

Overexpression of SHP: HepG2 cells were seeded at a density of 6×10^5 cells/well in 6-well plates and incubated in maintenance medium for 40–44 hours. PHHs were seeded at a density of 4×10^5 cells/well in 6-well plates and incubated in maintenance medium for 24–28 hours. Transfection was performed using Lipofectamine 3000 (Invitrogen) to deliver the expression plasmid into the cells.

Cell staining and quantification: Cells were fixed in 4% paraformaldehyde for 10 minutes, permeabilized by 0.01% Triton X-100, blocked with 3% BSA, and incubated with the indicated primary antibodies (against SHP) in 3% BSA overnight at 4°C. After washing, samples were incubated with the appropriate secondary antibody and DAPI (FP1490; ImmunoBioScience) for 1 hour at room temperature and then mounted in Fluoroshield with mounting media (ab104135, Abcam). For BODIPY staining, cells were washed twice with PBS, and fixed with 4% paraformaldehyde for 10 minutes. Followed by permeabilization with 0.01% Triton X-100 and two additional PBS washes, cells were incubated with 5 µg/mL BODIPY at 37 °C for 30 minutes, and stained with 1 µg/mL DAPI for 10 minutes at room temperature. Micrographs were acquired by confocal microscopy (FV3000, Olympus). The quantification of signals and the nuclear co-localization of all images were carried out using TissueQuest 7.1 software (TissueGnostics).

Immunoblotting: HepG2 cell lysate was placed in RIPA buffer containing a protease inhibitor cocktail, and centrifuged. The protein concentration of the resulting supernatant was determined using a protein assay kit (Bio-

Rad Laboratories, Hercules, CA, USA). Samples were mixed with SDS loading buffer, boiled, electrophoresed in SDS-PAGE gels, and then transferred to PVDF membranes. Membranes were blocked with blocking buffer for 1 h at room temperature and incubated with primary antibodies specific for human, which were listed in **the bellowing Table**. After washing, the membranes were incubated with horseradish peroxidase-conjugated secondary antibodies. Immunoreactive protein detection was performed with an enhanced chemiluminescence detection system (PerkinElmer, Waltham, MA, USA).

Table. List of antibodies used in experiments

**Antibody**	**Source**	**Brand, cat#**
Phospho-IKKα/β (ser176/180)	Rabbit	Cell Signaling, #2697
IKKα	Rabbit	Cell Signaling, #2682
IKKβ	Rabbit	Cell Signaling, #2370
Phospho-IκBα (ser32/36)	Mouse	Cell Signaling, #9246
IκBα	Mouse	Cell signaling, #4814
Phospho-NFκB (ser536)	Rabbit	ThermoFisher, MA5-15160
NFκB	Rabbit	Santa Cruz, sc-372
TLR2	Rabbit	Santa Cruz, sc-16240
TLR4	Goat	Santa Cruz, sc-10739
MYD88	Rabbit	Sigma, AB16527
NLRP3	Goat	Abcam, ab4207
Caspase I	Rabbit	Abcam, ab179515
IL1β	Goat	R&D system, AF-401-NA
Gasdermin D (GSDMD)	Rabbit	Cell signaling, #93709
β-actin	Mouse	Sigma, A2228
NR0B2	Rabbit	GeneTex, GTX54598
PKCζ	Mouse	Santa Cruz, sc-17781

Fig. S1: Algorism of study participants

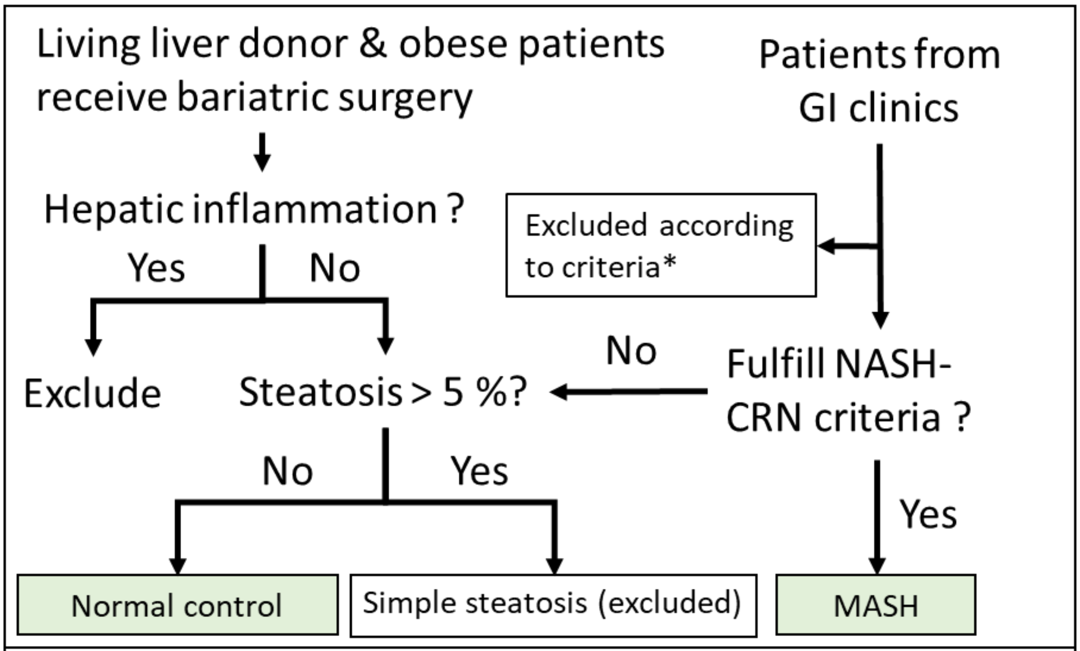

* Exclusion criteria:

- Other etiology of chronic hepatitis (HBV, HCV, AIH, PBC, hemochromatosis, Wilson disease, alcoholic hepatitis.)
- Active alcohol consumption (defined as excessive alcohol consumption within 5 years)
- Drugs that induce steatosis and/or influent bile acids or affect nature course of NASH
  - ✓ Screen by Livertox (https://livertox.nlm.nih.gov/index.html).
  - ✓ Drugs including ursodeoxycholic acid, fibrate, cholestyramine and pioglitazone within 1 month before biopsy.
- Extreme outlier: : total BA: > 26. mRNA: significant outlier.

Fig. S2: Ratios of nuclear SHP in patients with MASH according to NAS score and fibrosis stage. (A) Nuclear SHP ratios stratified by MASH risk: at-risk MASH (NAS ≥4 and fibrosis stage ≥2) vs. low-risk MASH (NAS ≤3 and fibrosis stage ≤2).

(B) Nuclear SHP ratios stratified by NAS score: high NAS (5–7 points) vs. low NAS (3–4 points).

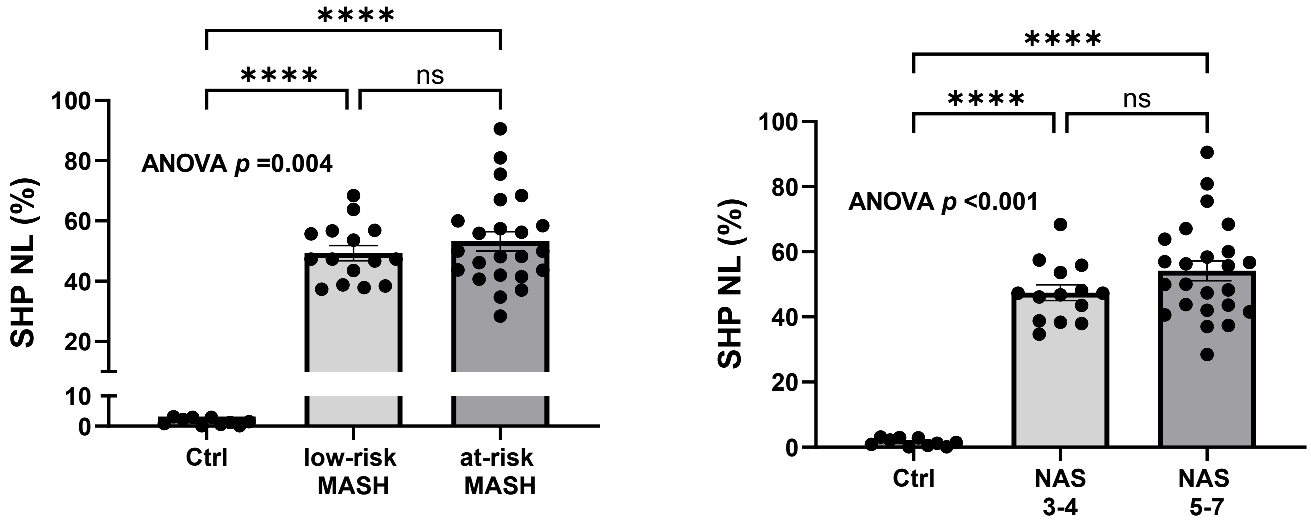

Fig. S3: Correlation between the nuclear SHP ratio and AI-assisted quantitative measurement of lipid droplet area in hepatocytes.

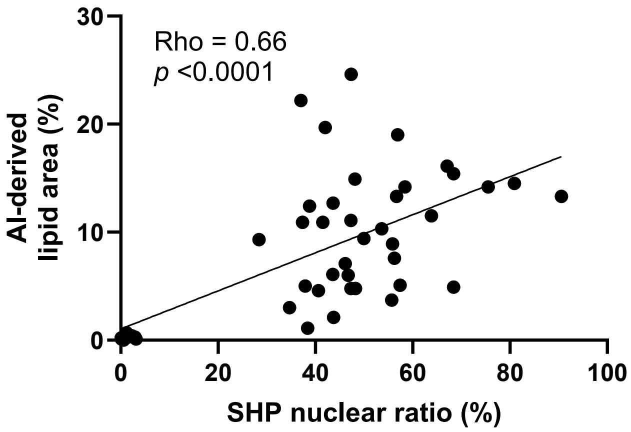

Fig. S4: Serum secondary bile acid, deoxycholic acid (DCA), is not associated with disease activity. (A) No significant differences were observed in levels of serum DCA species (unconjugated DCA, glyco-DCA, and tauro-DCA) across different pathological grades of disease activity. (B) Correlation matrix analysis showed no association between serum DCA levels and serological markers of hepatic inflammation.

(A)

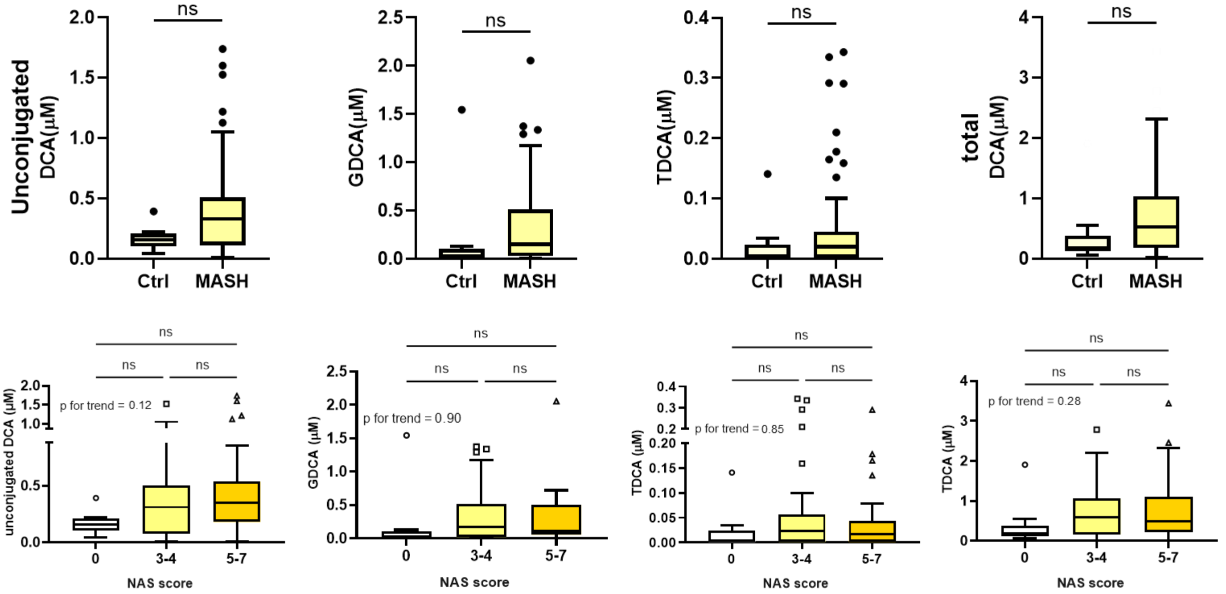

(B)

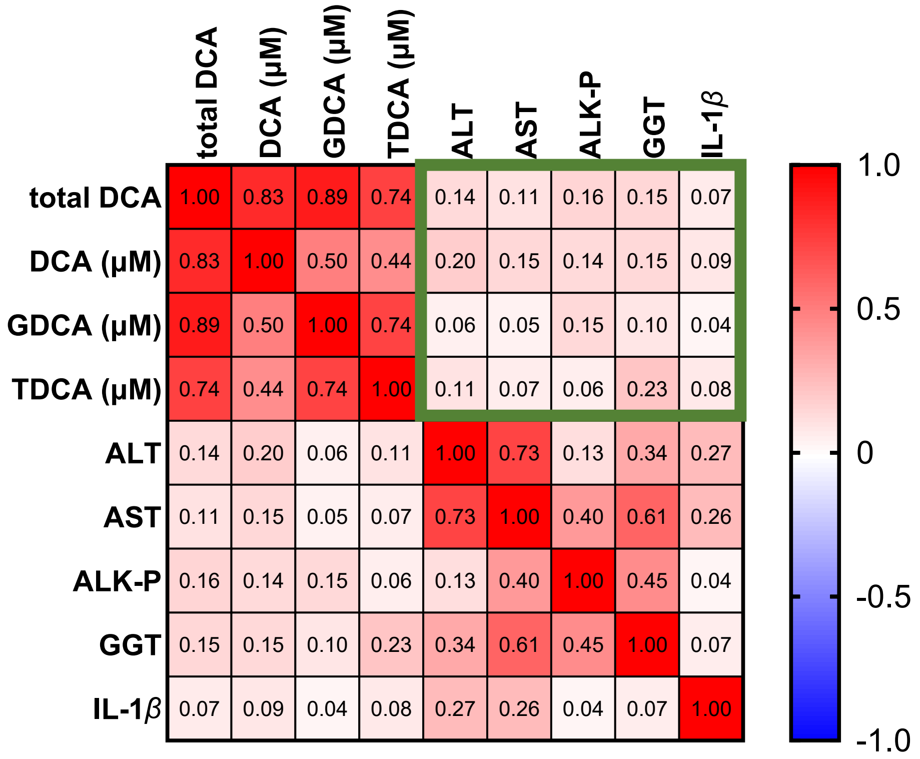

Fig. S5: The hepatic mRNA levels of *PEPCK* and *G6PC* were upregulated in patients with MASH, but progressively decreased as fibrosis stage advanced. *: $p < 0·05$ (Mann-Whitney U test), p for trend (ANOVA with polynomial contrast).

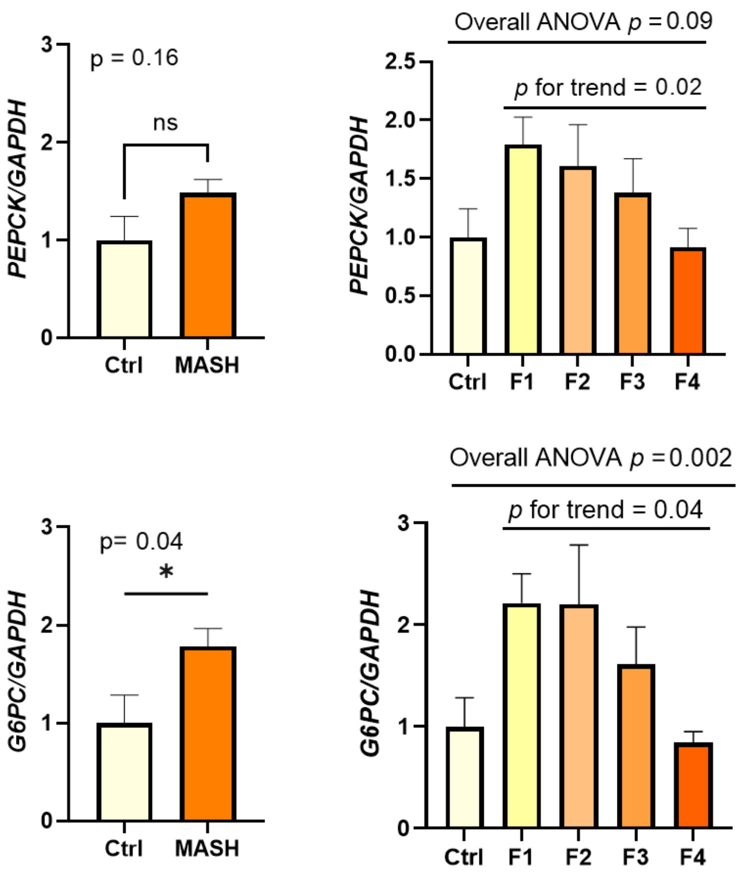

Fig. S6: *In vitro*, primary human hepatocytes (PHHs) exposed to palmitic acid (PA) and the inflammatory cytokine IL-1β—similar to chenodeoxycholic acid (CDCA)—elicited nuclear translocation of SHP. Inhibition of PKCζ attenuated SHP nuclear translocation induced by PA, IL-1β, and CDCA.

(A) Upper panel: Immunofluorescent staining of SHP (red) and nuclei (DAPI, blue) in PHHs treated with IL-1β, palmitic acid (PA), chenodeoxycholic acid (CDCA), or ddH_2O (control). Lower panel: Effects of PKCζ inhibition using its N-myristoylated pseudosubstrate inhibitor under the same stimulation conditions. (B) Quantification of nuclear SHP signal intensity in PHHs under the indicated stimulation conditions. Each experiment was performed in triplicate. Scale bar = 50 μm. *p < 0.001. *: $p < 0{\cdot}05$, **: $p < 0{\cdot}01$, ***: $p < 0{\cdot}001$ (Kruskal-Wallis test).

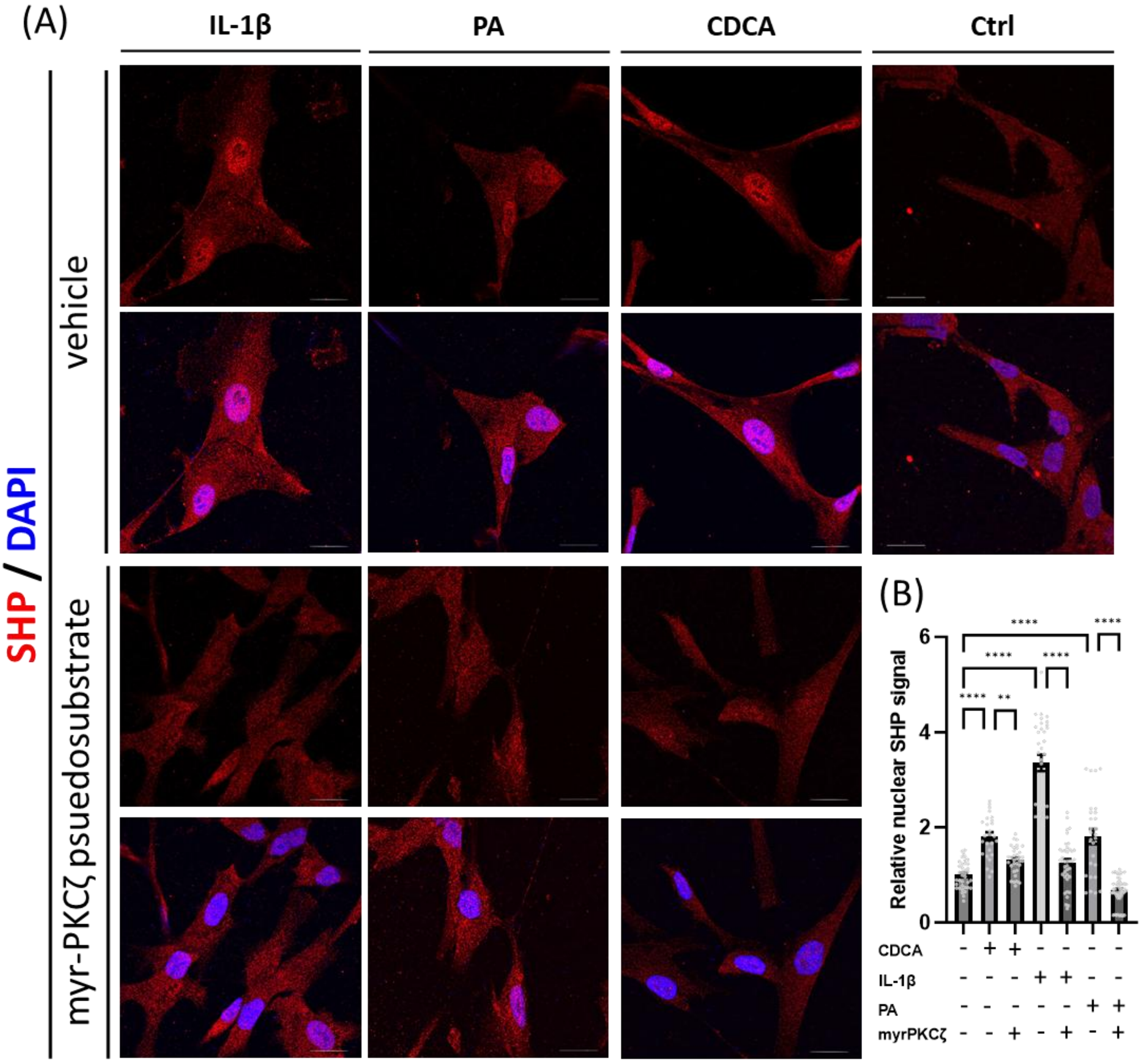

Fig. S7: Analysis of the GEO database identified two datasets with genome-wide expression profiles from liver-specific *SHP* knockdown in MASH-related animal models (GSE133566 and GSE38013). Under Western diet feeding, mice with SHP knockdown exhibited distinct hepatic gene expression clusters compared to wild-type controls. These differentially expressed genes were enriched in immune-related pathways, particularly those involved in innate immune responses.

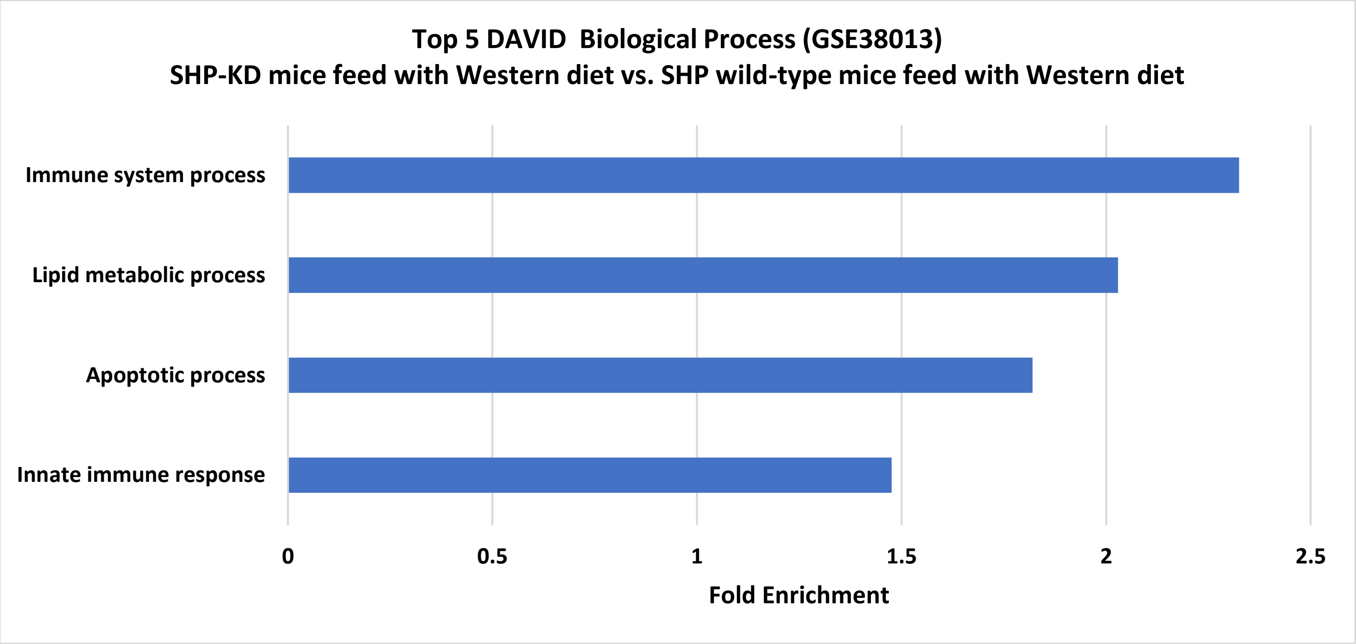

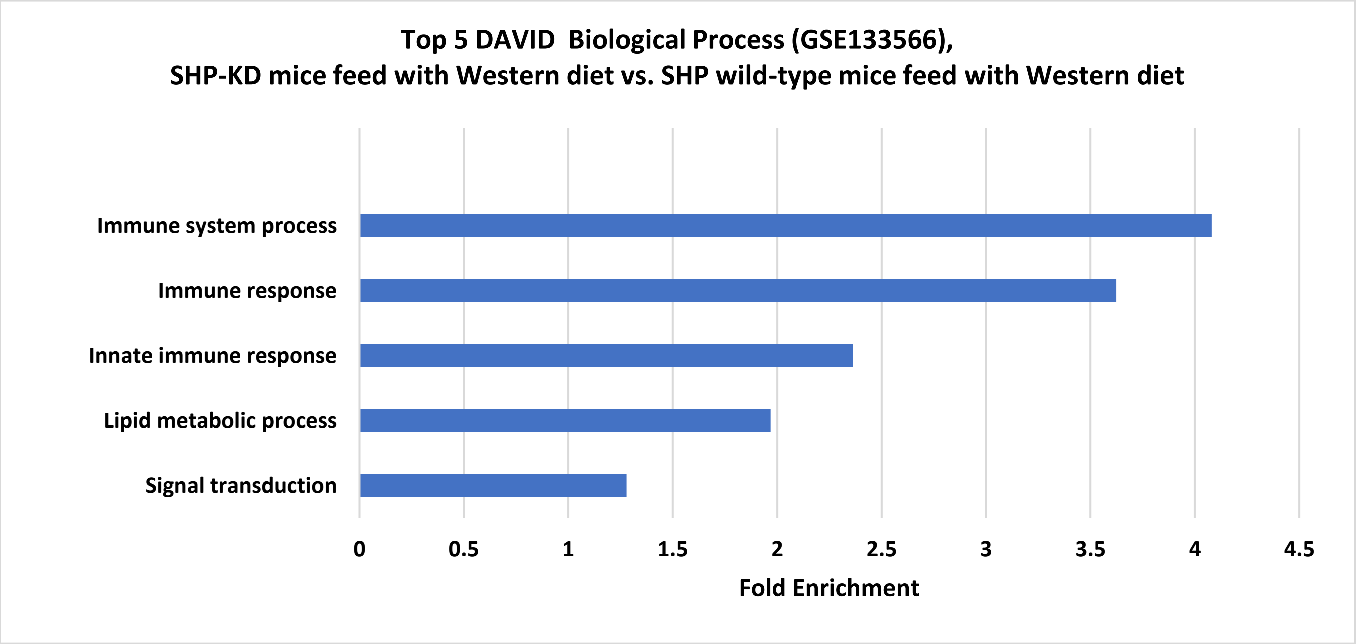

Fig. S8: *SHP* knockdown for long-term in HepG2 cells led to more pronounced increases in basal levels of innate immune-related proteins, regardless of IL-1β treatment. (Comparison of lanes 1 and 3 represent treatment without IL-1β for 6-hour, while lanes 5 and 7 correspond to 24-hour).

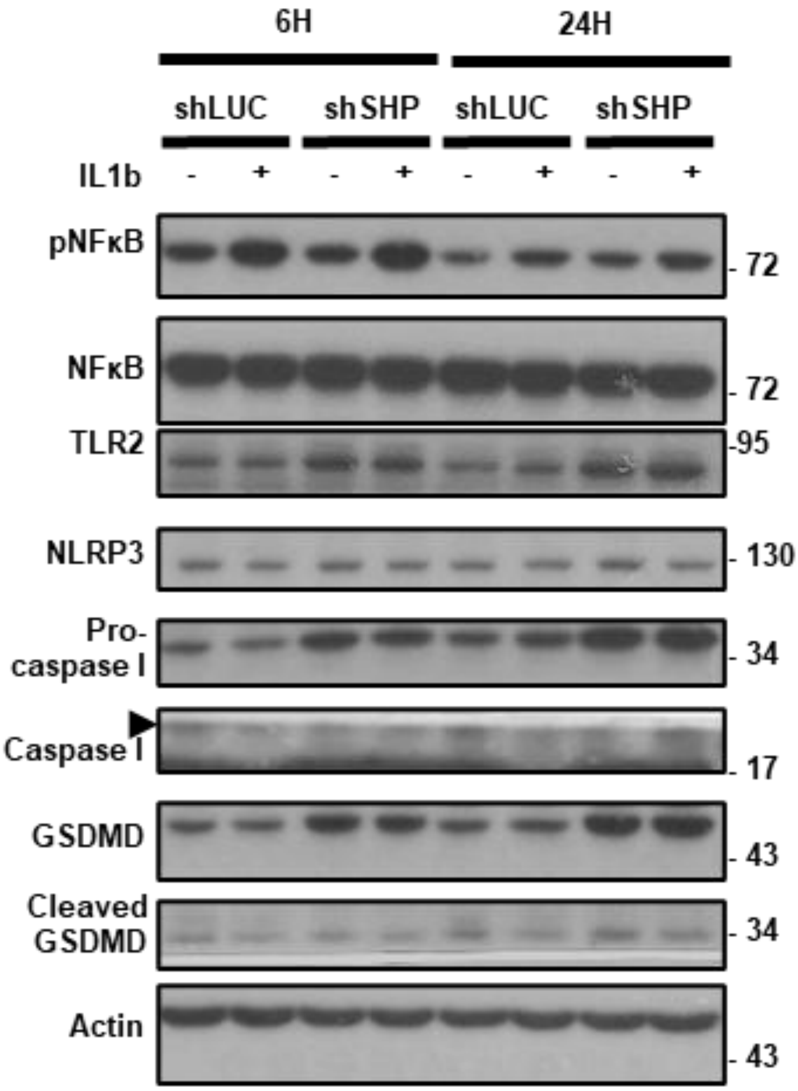

Fig. S9: Western blot quantification of innate immune–related proteins in *SHP*-overexpressing HepG2 cells. *: $p < 0{\cdot}05$, **: $p < 0{\cdot}01$, ***: $p < 0{\cdot}001$ (Kruskal-Wallis test).

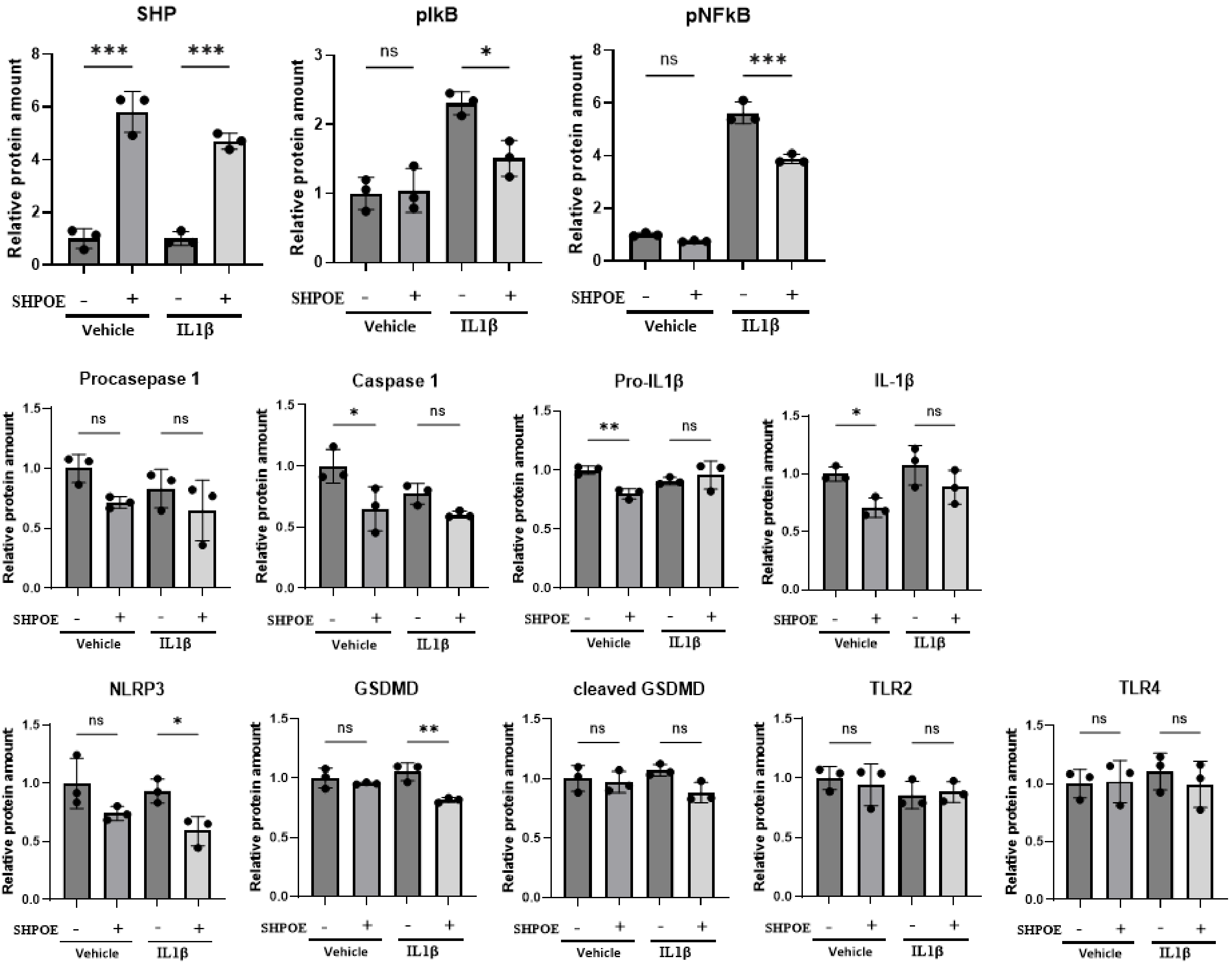

Fig. S10: mRNA levels of selected innate inflammatory genes following *SHP* overexpression and IL-1β treatment. *: $p < 0{\cdot}05$, **: $p < 0{\cdot}01$, ***: $p < 0{\cdot}001$ (Kruskal-Wallis test).

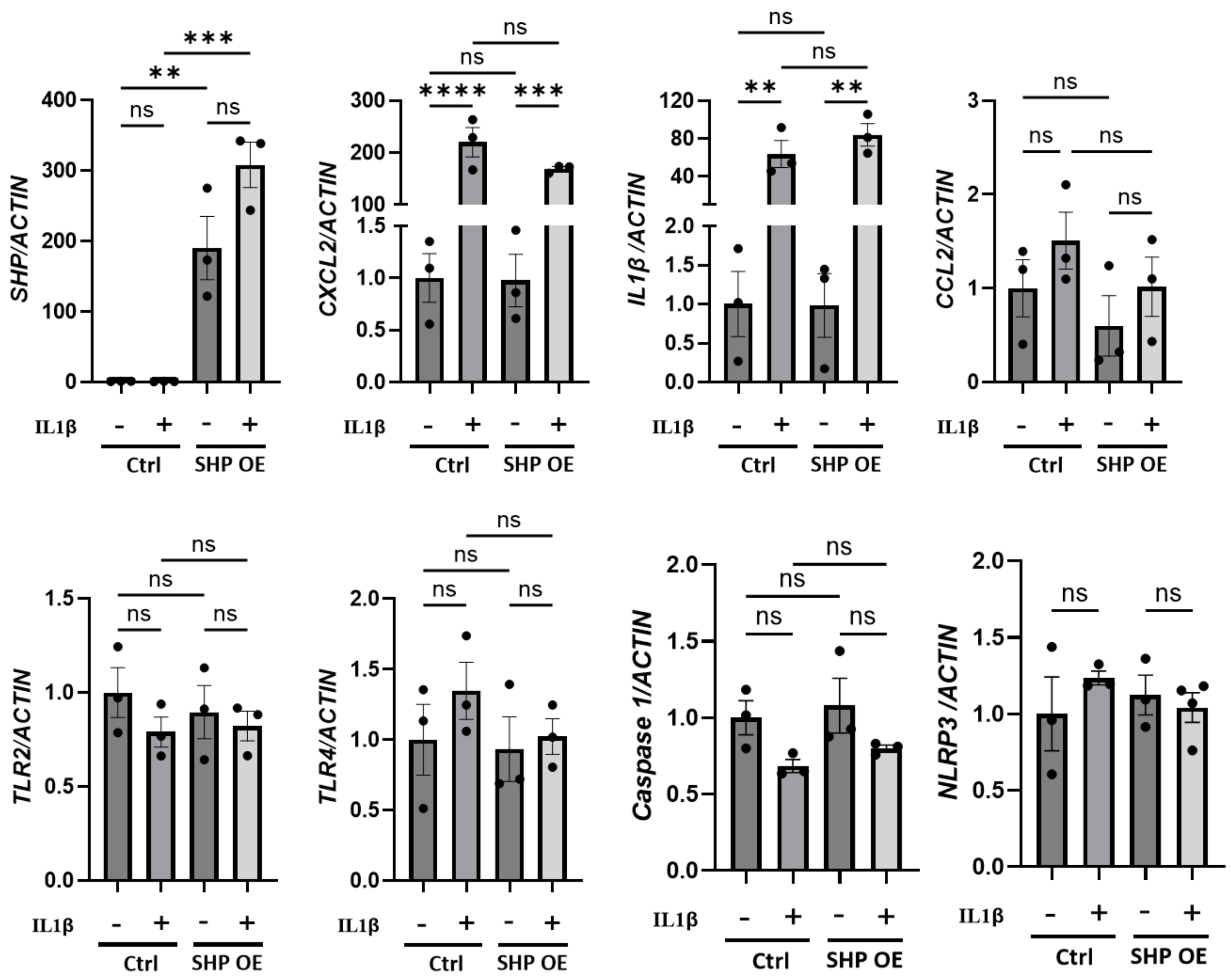

Table S1. Primer sequences designed for qPCR

Genes	Species	Forward primer	Reverse primer
CYP7A1	Human	5'- AAATCTACCCAGACCCTTTG-3'	5'- TTCCAGGACATATTGTAGCTC -3'
CYP8B1	Human	5'- CAGTAGAGACATTGCTGTTC-3'	5'- TATGATACAAATGGTTGCTGC-3'
CYP7B1	Human	5'- AGCACATCATTTAGGCTTTC-3'	5'- GCAGAAGATAATACATTGCCC-3'
CYP27A1	Human	5'- ATACGGATGCTTTCAATGAG-3'	5'- CGAACAGGATGTAGCAAATAG-3'
BACS	Human	5'- CGGTACTTGTGTAACATTCC-3'	5'- GACTTCCCAGATCCGAATAG-3'
BAAT	Human	5'- ATAACTATGAAGACCTGCCC-3'	5'- AAGACCTTTGGATGTCTCAG-3'
NTCP	Human	5'- CTTTCTGCTGGGTTATGTTC-3'	5'- CTGGAAAATCATGTAGAGGAG-3'
OATP1B1	Human	5'- GGTTGTTTAAAGGAATCTGGG-3'	5'- TGGACCAATCATTGCTATTG-3'
OATP1B3	Human	5'- CTGAGCACTATCAGAATAACTC-3'	5'- TTCAGCACATGCAATGATAG-3'
BSEP	Human	5'- CAGATTACAAATGAAGCCCTC-3'	5'- TCCATATCTGTAGGAAGCAG-3'
MDR2	Human	5'- AAATTGCTGATCTCCTTTGC-3'	5'- GATAGCTGTCCGTACTTTTAC-3'
MDR3	Human	5'- GAGGTCAAAAACAGAGGATTG-3'	5'- CCTTTTCACTTTCAGTATCCAG-3'
OSTA	Human	5'-TATTCCTCTAAAACCAGGTCTC-3'	5'-TACAGCATCCTTTCATTGTC-3'
OSTB	Human	5'-GCAGAAAAGAAAAGATGCAG-3'	5'-CTTAGGTTGTTTAGGCTGTTG-3'
SREBP1C	Human	5'-CTTCCCAGCCCCTCAGATA-3' -3'	5'-TGTGACTGGCTCACCGTAGA-3'
ACC	Human	5'- CAGTGAAGGCTTATGTTTGG-3'	5'- CGTCATATGGATGATGGAATC-3'
PPARG	Human	5'-AAAGAAGCCGACACTAAACC-3'	5'-CTTCCATTACGGAGAGATCC-3'
G6PC	Human	5'-ACTGTGCATACATGTTCATC-3'	5'-TGAATGTTTTGACCTAGTGC-3'
PEPCK	Human	5'-ATTCTGGGTATAACCAACCC-3'	5'-GTTGATGGCCCTTAAATGAC-3'
β-actin	Human	5'-CTGGACTTCGAGCAAGAGATG-3'	5'-TGATGGAGTTGAAGGTAGTTTCG-3'
NR0B2	Human	5'-CTTCAACCCCGATGTGCCAG-3'	5'-GGTCGGAATGGACTTGAGGG-3'

Table S2: Baseline characteristics of the study cohort. Data are presented as median (range) for continuous variables and number (percentage) for categorical variables. Pearson's Chi-square test was used for categorical comparisons, and the Kruskal–Wallis test was applied for non-parametric continuous data.
*: p < 0.05; **: p < 0.01; ***: p < 0.001. †Data available for only 3 patients.

		Control (n=10)	MASH (n=68)	p value
Age		35 (20-41)	54 (23 - 82)	<0.001***
Gender	M vs. F	6 (60.0%) vs. 4 (40.0%)	42 (61.8%) vs. 26 (38.2%)	0.915
BW		67.7 (54.0-117.3)	80.1 (48.6 - 130.7)	0.018*
BMI (kg/m2)		23.06 (20.03-37.31)	28.9 (20.9 - 41.5)	0.002**
HTN	N vs. Y	10 (100.0%) / 0 (0.0%)	42 (61.8%) / 26 (38.2%)	0.017*
DM	N vs. Y	10 (100.0%) / 0 (0.0%)	36 (52.9%) / 32 (47.1%)	0.005**
Dyslipidemia	N vs. Y	10 (100.0%) / 0 (0.0%)	46 (67.6%) / 22 (32.4%)	0.03*
CAP (dB/M)			309 (237 - 400)	
LSM (kPA)			8.1 (3.3 - 49.6)	
ARFI (m/s)			1.32 (0.86 - 3.46)	
ALT (U/L)		14 (9-23)	78 (11 - 210)	<0.001***
AST (U/L)		19 (13-33)	53 (22 - 194)	<0.001***
ALP (U/L)		58 (46-96)	77 (39 - 187)	0.014*
Γ-GT (U/L)		11 (9-35)	55 (9 - 474)	<0.001***
Alb (g/dL)		4.6 (4.1-5.0)	4.7 (3.8 - 5.4)	0.75
Bilirubin-T (mg/d)		0.6 (0.2-2.1)	0.6 (0.3 - 3.6)	0.71
PT (INR)		1.09 (0.96-1.23)	1.03 (0.90 - 1.29)	0.06
Cr (mg/dL)		0.74 (0.52-0.95)	0.78 (0.39 - 1.19)	0.85
LDL (mg/dL)		84 (41-114)	113 (52 - 218)	0.07
HDL (mg/dL)		40 (14-53)	46 (22 - 139)	0.29
TG (mg/dL)		71 (45-105)	127 (63 - 708)	<0.001***
Cholesterol (mg/dL)		151 (63-225)	168 (112 - 263)	0.1
Glucose AC (mg/dL)		90 (75-103)	111 (67 - 227)	<0.001***
Insulin (uU/ml)		9.1 (6.0-19.7) †	17.20 (1.60 - 119.31)	0.06
HOMA-IR		2.07 (1.38-5.01) †	4.94 (0.54 - 32.99)	0.044*
Ferritin (ng/mL)		129.66 (51.43-887.50)	322.6 (21.7 - 2980.0)	0.3
NAS score		0	4 (3-7)	
Steatosis	1/2/3	0/0/0	28 (41.2%) / 25 (36.8%) / 15 (22.1%)	
Ballooning	1/2	0/0	49 (72.1%) / 17 (25.0%)/ 2 (2.9%)	
Lobular inflammation	1/2/3	0/0/0	47 (69.1%) / 21 (30.9%)	
Fibrosis stage	1/2/3/4	0/0/0	26 (38.2%) / 10 (14.7%) / 19 (27.9%) / 13 (19.1%)	

Table S3: Linear regression analysis of hepatic IL-1β mRNA expression. Results are presented as β coefficients with corresponding 95% confidence intervals. CMRF: Cardiometabolic risk factors used in the diagnosis of MASLD. Data are presented as β coefficients and 95% confidence interval (95% CI).

IL-1B mRNA regression analysis								
	Univariate analysis		Multivariable analysis					
			Model 1		Model 2		Model 3	
	β Coefficients (95% CI)	p	β Coefficients (95% CI)	p	β Coefficients (95% CI)	p	β Coefficients (95% CI)	p
Age	-0.01 (-0.02 - 0.01)	0.28					-0.01 (-0.03 - 0.01)	0.37
Gender	0.16 (-0.17 - 0.49)	0.34					0.11 (-0.38 - 0.60)	0.65
SHP nuclear ratio (%)	- 0.001 (-0.011 - 0.008)	0.75	-0.01 (- -0.01 - 0.01)	0.50			-0.003 (-0.019 - 0.012)	0.66
NAS-CRN Grade:								
Steatosis	0.15 (-0.02 - 0.32)	0.08	0.15 (- 0.15 - 0.45)	0.33	0.11 (-0.07 - 0.30)	0.24	0.15 (-0.16 - 0.45)	0.34
Lobular inflammation	0.05 (-0.20 - 0.29)	0.70						
Ballooning	-0.03 (-0.29 - 0.23)	0.81						
NAS score	0.04 (-0.05 - 0.13)	0.33						
Fibrosis	0.003 (-0.12 - 0.13)	0.96						
ALT	0.003 (0.0006 - 0.0062)	0.02	-0.0003 (- 0.010 - 0.004)	0.35	0.000 (-0.004 - 0.005)	0.87	-0.003 (-0.010 - 0.004)	0.38
AST	0.005 (0.008 - 0.009)	0.02	0.01 (- 0.0004 - 0.019)	0.06	0.003 (-0.003 - 0.009)	0.34	0.010 (-0.001 - 0.020)	0.06
ALP	0.001 (-0.0-055 - 0.0076)	0.75						
GGT	0.001 (-0.0013 - 0.0024)	0.56						
Ferritin	-0.00005 (-0.0003 - 0.0003)	0.75						
LDL	0.003 (- 0.0013 - 0.0067)	0.18						
TG	-0.0005 (-0.002 - 0.001)	0.54						
Cholesterol	0.0004 (-0.0004 - 0.008)	0.08						
HbA1C	-0.004(-0.17 - 0.16)	0.96						
HOMA-IR	-0.01 (-0.03 - 0.02)	0.56						
BMI	0.0002 (-0.03 - 0.03)	0.99						
CMRF	-0.10 (-0.22 - 0.02)	0.10						
Dyslipidemia under statin	-0.45 (-0.80 - -0.11)	0.01	-0.61 (- -0.61 - 0.07)	0.08	-0.451 (-0.802 - -0.101)	**0.01**	-0.61 (-1.32 - 0.10)	0.09
HTN	-0.11 (-0.45 - 0.23)	0.51						
DM	-0.07 (-0.40 - 0.25)	0.66						

Table S4: Medications related to cardiometabolic risk factors (CMRFs) used by participants in the study cohort. Data are presented as number and percentage (%)

	Medication Type	N	(%)	SHP NL (%)
Medication for T2DM	Metformin only	3	9.4%	52.3%
	Metformin + SU	4	12.5%	
	Metformin + SU + DPP4	7	21.9%	
	Metformin + SU + SGLT2	1	3.1%	
	Metformin + DPP4	4	12.5%	
	Metformin + DPP4 + α-glucosidate inhibitor	1	3.1%	
	Metformin + insulin	2	6.3%	
	DPP4	1	3.1%	
	No drug	9	28.1%	51.1%
Medication for dyslipidemia	Statin	12	54.5%	57.5%
	Statin combine Ezetimab	4	18.2%	
	Fibrate	0	0.0%	49.9%
	No drug	6	27.3%	

Table S5: Correlation between nuclear SHP ratio and bile acid–related markers.
Rho: Spearman correlation coefficient.*: $p < 0.05$; **: $p < 0.01$; ***: $p < 0.001$.

	**Rho (p value)**
**BAs related genes**	
CYP7A1	0.52 (<0.001) ***
CYP8B1	-0.05 (0.72)
CYP7B1	0.29 (0.04) *
CYP27A1	0.19 (0.19)
BACS	-0.11 (0.47)
BAAT	0.24 (0.10)
NTCP	0.31 (0.03) *
OATP1B1	0.31 (0.03) *
OATP1B3	0.34 (0.02) *
BSEP	0.30 (0.04) *
MDR2	0.33 (0.02) *
MDR3	0.36 (0.01) *
OSTα	- 0.26 (0.07)
OSTβ	0.17 (0.25)
**BAs**	
Total BA	0.22 (0.14)
Conjugated BA	0.19 (0.19)
Unconjugated BA	0.24 (0.10)
Primary BA	0.22 (0.13)
Secondary BA	0.14 (0.36)
**Primary BAs**	
CA	0.21 (0.15)
CDCA	0.20 (0.17)
GCA	0.32 (0.03) *
GCDCA	0.20 (0.19)
TCA	0.17(0.25)
TCDCA	0.14 (0.34)
**Secondary BAs**	
LCA	0.14 (0.36)
DCA	0.20 (0.17)
UDCA	-0.08 (0.60)
GLCA	-0.06 (0.70)
GDCA	0.13 (0.37)
GUDCA	-0.03 (0.83)

TLCA	-0.15 (0.32)
TDCA	0.05 (0.74)
TUDCA	0.14 (0.34)
