# Supplementary material for: Enhanced nuclear localization of small heterodimer partner in metabolic dysfunction-associated steatohepatitis
Source: JHEP Rep. 2025 Oct 4;8(1):101616. doi: 10.1016/j.jhepr.2025.101616 (PMC12721038; doi:10.1016/j.jhepr.2025.101616)
Supplement: Multimedia component 2 [file mmc2.docx]

**JHEP Reports**

**CTAT methods**

Tables for a “Complete, Transparent, Accurate and Timely account” (CTAT) are now mandatory for all revised submissions. The aim is to enhance the reproducibility of methods.

- Only include the parts relevant to your study
- Refer to the CTAT in the main text as ‘Supplementary CTAT Table’
- Do not add subheadings
- Add as many rows as needed to include all information
- Only include one item per row

**If the CTAT form is not relevant to your study, please outline the reasons why:**

|  |
| --- |

- 1. **Antibodies**

| **Name** | **Citation** | **Supplier** | **Cat no.** | **Clone no.** |
| --- | --- | --- | --- | --- |
| **Antibody** | **Source** | **Brand, cat#** |  |  |
| Phospho-IKKα/β (ser176/180) | Rabbit | Cell Signaling, | #2697 | 16A6 |
| IKKα | Rabbit | Cell Signaling | #2682 | — |
| IKKβ | Rabbit | Cell Signaling | #2370 | 2C8 |
| Phospho-IκBα (ser32/36) | Mouse | Cell Signaling | #9246 | 5A5 |
| IκBα | Mouse | Cell Signaling | #4814 | L35A5 |
| Phospho-NFκB (ser536) | Rabbit | ThermoFisher, | MA5-15160 | EP229 |
| NFκB | Rabbit | Santa Cruz | sc-372 | C-20 |
| TLR2 | Rabbit | Santa Cruz | sc-16240 | H-175 |
| TLR4 | Goat | Santa Cruz | sc-10739 | N/A (polyclonal) |
| MYD88 | Rabbit | Sigma | AB16527 | — |
| NLRP3 | Goat | Abcam | ab4207 | — |
| Caspase I | Rabbit | Abcam | ab179515 | EPR16887 |
| IL1β | Goat | R&D system | AF-401-NA | Polyclonal |
| Gasdermin D (GSDMD) | Rabbit | Cell signaling | #93709 | E1Z3S |
| β-actin | Mouse | Sigma | A2228 | AC-15 |
| NR0B2 | Rabbit | GeneTex, | GTX54598 | — |
| PKCζ | Mouse | Santa Cruz | sc-17781 | C-20 |

- 1. **Cell lines**

| **Name** | **Citation** | **Supplier** | **Cat no.** | **Passage no.** | **Authentication test method** |
| --- | --- | --- | --- | --- | --- |
| HepG2 | Human carcinoma | ATCC HB 8065 | Lot-02436 | 85 | STR profiling |
| Primary human hepatocytes (PHHs) | Human Hypatocytes | ScienCell | Catalog #5200 | **-** | STR profiling |

- 1. **Organisms**

| **Name** | **Citation** | **Supplier** | **Strain** | **Sex** | **Age** | **Overall n number** |
| --- | --- | --- | --- | --- | --- | --- |
| **N/A** | | | | | | |

- 1. **Sequence based reagents**

| **Name** | **Forward primer** | **Reverse primer** | **Supplier** |
| --- | --- | --- | --- |
| CYP7A1 | 5’- AAATCTACCCAGACCCTTTG-3’ | 5’- TTCCAGGACATATTGTAGCTC  -3’ | Sigma-Aldrich |
| CYP8B1 | 5’- CAGTAGAGACATTGCTGTTC-3’ | 5’- TATGATACAAATGGTTGCTGC-3’ | Sigma-Aldrich |
| CYP7B1 | 5’- AGCACATCATTTAGGCTTTC-3’ | 5’- GCAGAAGATAATACATTGCCC-3’ | Sigma-Aldrich |
| CYP27A1 | 5’- ATACGGATGCTTTCAATGAG-3’ | 5’- CGAACAGGATGTAGCAAATAG-3’ | Sigma-Aldrich |
| BACS | 5’- CGGTACTTGTGTAACATTCC-3’ | 5’- GACTTCCCAGATCCGAATAG-3’ | Sigma-Aldrich |
| BAAT | 5’- ATAACTATGAAGACCTGCCC-3’ | 5’- AAGACCTTTGGATGTCTCAG-3’ | Sigma-Aldrich |
| NTCP | 5’- CTTTCTGCTGGGTTATGTTC-3’ | 5’- CTGGAAAATCATGTAGAGGAG-3’ | Sigma-Aldrich |
| OATP1B1 | 5’- GGTTGTTTAAAGGAATCTGGG-3’ | 5’- TGGACCAATCATTGCTATTG-3’ | Sigma-Aldrich |
| OATP1B3 | 5’- CTGAGCACTATCAGAATAACTC-3’ | 5’- TTCAGCACATGCAATGATAG-3’ | Sigma-Aldrich |
| BSEP | 5’- CAGATTACAAATGAAGCCCTC-3’ | 5’- TCCATATCTGTAGGAAGCAG-3’ | Sigma-Aldrich |
| MDR2 | 5’- AAATTGCTGATCTCCTTTGC-3’ | 5’- GATAGCTGTCCGTACTTTTAC-3’ | Sigma-Aldrich |
| MDR3 | 5’- GAGGTCAAAAACAGAGGATTG-3’ | 5’- CCTTTTCACTTTCAGTATCCAG-3’ | Sigma-Aldrich |
| OSTA | 5’-TATTCCTCTAAAACCAGGTCTC-3’ | 5’-TACAGCATCCTTTCATTGTC-3’ | Sigma-Aldrich |
| OSTB | 5’-GCAGAAAAGAAAAGATGCAG-3’ | 5’-CTTAGGTTGTTTAGGCTGTTG-3’ | Sigma-Aldrich |
| SREBP1C | 5’-CTTCCCAGCCCCTCAGATA-3’ -3’ | 5’-TGTGACTGGCTCACCGTAGA-3’ | Sigma-Aldrich |
| ACC | 5’- CAGTGAAGGCTTATGTTTGG-3’ | 5’- CGTCATATGGATGATGGAATC-3’ | Sigma-Aldrich |
| PPARG | 5’-AAAGAAGCCGACACTAAACC-3’ | 5’-CTTCCATTACGGAGAGATCC-3’ | Sigma-Aldrich |
| G6PC | 5’-ACTGTGCATACATGTTCATC-3’ | 5’-TGAATGTTTTGACCTAGTGC-3’ | Sigma-Aldrich |
| PEPCK | 5’-ATTCTGGGTATAACCAACCC-3’ | 5’-GTTGATGGCCCTTAAATGAC-3’ | Sigma-Aldrich |
| β-actin | 5’-CTGGACTTCGAGCAAGAGATG-3’ | 5’-TGATGGAGTTGAAGGTAGTTTCG-3’ | Sigma-Aldrich |
| NR0B2 | 5’-CTTCAACCCCGATGTGCCAG-3’ | 5’-GGTCGGAATGGACTTGAGGG-3’ | MISSION BIOTECH |

- 1. **Biological samples**

| **Description** |  | **Source** | **Identifier** |
| --- | --- | --- | --- |
| Liver tissue |  | Human | Not applicable |
| Blood serum |  | Human | Not applicable |

- 1. **Deposited data**
- The datasets generated during and/or analyzed during the current study are available from the corresponding author on reasonable request.

- 1. **Software**

| **Software name** | **Manufacturer** | **Version** |
| --- | --- | --- |
| TissueQuest | TissueGnostics | 7.1 |
| SPSS | IBM | 25 |

- 1. **Other (*e.g*. drugs, proteins, vectors etc.)**

| SHP overexpression | pcDNA3.4-NR0B2-Flag | LEADGENE BIO |
| --- | --- | --- |
| SHP knockdown | Lentivirus contained NR0B2-specific small hairpin RNA | (shRNA, CACATTGGACTTCCTTGGTTT) |
| Liquid chromatography–mass spectrometry | Cat. NO: API 5000 tandem mass spectrometer | Company: SCIEX |
| human IL1β Recombinant protein | 200-01B | peprotech |
| BSA - Palmitate Saturated fatty acid complexes (5mM) | 29558 | cayman |

- 1. **Please provide the details of the corresponding methods author for the manuscript:**

| **Name:** Shih-Chieh Chien **Affiliation:** Department of Internal Medicine, National Cheng Kung University Hospital, College of Medicine, National Cheng Kung University, Tainan, Taiwan **Email:** slamdunk9031137@gmail.com |
| --- |

**2.0 Please confirm for randomised controlled trials all versions of the clinical protocol are included in the submission. These will be published online as supplementary information.**

| **N/A** |
| --- |
